# Histone macroH2A1.2 promotes metabolic health and leanness by inhibiting adipogenesis

**DOI:** 10.1186/s13072-016-0098-9

**Published:** 2016-10-25

**Authors:** Valerio Pazienza, Concetta Panebianco, Francesca Rappa, Domenico Memoli, Michela Borghesan, Sara Cannito, Asami Oji, Giuseppe Mazza, Domenico Tamburrino, Giuseppe Fusai, Rosario Barone, Giulia Bolasco, Francesc Villarroya, Joan Villarroya, Kiyotaka Hatsuzawa, Francesco Cappello, Roberta Tarallo, Tomoko Nakanishi, Manlio Vinciguerra

**Affiliations:** 1 Gastroenterology Unit, IRCCS “Casa Sollievo della Sofferenza” Hospital, 71013 San Giovanni Rotondo, Italy; 2 Department of Experimental Biomedicine and Clinical Neurosciences, Section of Human Anatomy, University of Palermo, 90127 Palermo, Italy; 3 Department of Legal, Society and Sport Sciences, University of Palermo, 90133 Palermo, Italy; 4 Euro-Mediterranean Institute of Science and Technology (IEMEST), 90146 Palermo, Italy; 5 Laboratory of Molecular Medicine and Genomics, Department of Medicine, Surgery and Dentistry ‘Schola Medica Salernitana’, University of Salerno, 84081 Baronissi, SA Italy; 6 Institute for Liver and Digestive Health, University College London (UCL), Royal Free Hospital, London, NW3 2PF UK; 7 Research Institute for Microbial Diseases, Osaka University, Suita, Osaka, 5650871 Japan; 8 Centre for HPB Surgery and Liver Transplantation, Royal Free Hospital, London, NW3 2QG UK; 9 Mouse Biology Unit, European Molecular Biology Laboratory (EMBL), 00015 Monterotondo, Italy; 10 Departament de Bioquimica i Biologia Molecular, Institut de Biomedicina de la Universitat de Barcelona (IBUB), and CIBER Fisiopatologia de la Obesidad y Nutricion, University of Barcelona, Barcelona, 08007 Spain; 11 Centro de Investigación Biomédica en Red Fisiopatología de la Obesidad y Nutrición (CIBEROBN) ISCIII, Madrid, Spain; 12 Faculty of Medicine, Tottori University, Yonago, 683-8503 Japan; 13 The Institute of Medical Sciences, University of Tokyo, Tokyo, 108-8639 Japan; 14 Center for Translational Medicine (CTM), International Clinical Research Center (ICRC), St. Anne’s University Hospital, Brno, 656 91 Czech Republic

**Keywords:** Histone variants, macroh2a1.2, Adipose tissue, Obesity

## Abstract

**Background:**

Obesity has tremendous impact on the health systems. Its epigenetic bases are unclear. MacroH2A1 is a variant of histone H2A, present in two alternatively exon-spliced isoforms macroH2A1.1 and macroH2A1.2, regulating cell plasticity and proliferation, during pluripotency and tumorigenesis. Their role in adipose tissue plasticity is unknown.

**Results:**

Here, we show evidence that macroH2A1.1 protein levels in the visceral adipose tissue of obese humans positively correlate with BMI, while macroH2A1.2 is nearly absent. We thus introduced a constitutive GFP-tagged transgene for macroH2A1.2 in mice, and we characterized their metabolic health upon being fed a standard chow diet or a high fat diet. Despite unchanged food intake, these mice exhibit lower adipose mass and improved glucose metabolism both under a chow and an obesogenic diet. In the latter regimen, transgenic mice display smaller pancreatic islets and significantly less inflammation. MacroH2A1.2 overexpression in the mouse adipose tissue induced dramatic changes in the transcript levels of key adipogenic genes; genomic analyses comparing pre-adipocytes to mature adipocytes uncovered only minor changes in macroH2A1.2 genomic distribution upon adipogenic differentiation and suggested differential cooperation with transcription factors. MacroH2A1.2 overexpression markedly inhibited adipogenesis, while overexpression of macroH2A1.1 had opposite effects.

**Conclusions:**

MacroH2A1.2 is an unprecedented chromatin component powerfully promoting metabolic health by modulating anti-adipogenic transcriptional networks in the differentiating adipose tissue. Strategies aiming at enhancing macroH2A1.2 expression might counteract excessive adiposity in humans.

**Electronic supplementary material:**

The online version of this article (doi:10.1186/s13072-016-0098-9) contains supplementary material, which is available to authorized users.

## Background

The current pandemic in obesity/metabolic syndrome (with 30–50% of the overall population affected in the Western world) is a risk factor for many types of diseases, including cardiovascular diseases and cancer. Epigenetic mechanisms of nuclear chromatin remodeling are increasingly recognized as crucial factors in the pathophysiology of obesity and related complications [1]. In fact, metabolic alterations in peripheral tissues are triggered at the cellular level by changes in gene transcriptional patterns dependent on the degree of nuclear chromatin compaction. The latter is regulated at several levels, allowing transcriptional plasticity. For instance, epigenetic marks such as DNA methylation are intensely investigated for their causal and associative role in the determination of body mass index (BMI) [2–4]. A recently emerged alternative mechanism of transcriptional plasticity is the replacement of canonical histones, around which DNA is wrapped (H2A, H2B, H3 and H4), with the incorporation of histone variants, mostly of histones H2A or H3 [5–7]. The histone variant of H2A, known as macroH2A1, is believed to act as a strong transcriptional modulator that can either repress transcription or activate it in response to as yet undefined nutrients or growth signals [8–13]. The impact of macroH2A1 on transcriptional processes has now come to take a center stage in the plasticity of stem cell differentiation and in the pathogenesis of a growing number of cancer types [14–17]. MacroH2A1 is composed of a domain displaying 66% homology with histone H2A, and a domain called macro that is conserved in multiple functionally unrelated proteins throughout the animal kingdom and that can bind in vitro with tight affinity ADP-ribose-like metabolites, providing a direct molecular interaction between intermediate metabolism and the chromatin, whereby a metabolite can tweak gene expression in vitro [18]. MacroH2A1 is in turn present in two alternatively exon-spliced isoforms, macroH2A1.1 and macroH2A1.2, which differ for a few amino acids [18]. Whether these two isoforms play different roles in cell plasticity is debated and context dependent; however, most reports support a pro-differentiation role for macroH2A1.1 and an anti-differentiation and pro-proliferative role for macroH2A1.2 [14–17, 19, 20]. Mice models, knockout (KO) for the whole macroH2A1 gene, have been reported. In KO mice generated in the pure C57BL/6 J background, modest developmental changes in macroH2A1-mediated gene regulation under a standard diet, and a very mild systemic protection against obesity upon a high fat regimen, were observed [21, 22]. By contrast, in KO mice for macroH2A1 generated in a mixed background a variable hepatic lipid accumulation in 50% of the females has been described, without changes in body weight [23]. Therefore, despite compelling in vitro evidence that macroH2A1 modulates gene expression programs involved in cell metabolism, proliferation and differentiation, the existing evidence for its role at the organism level upon nutritional stress, especially during fat accumulation obesity, is controversial. Moreover, data deriving from KO approaches might be often influenced by functional redundancy or compensatory effects between the isoforms. Under a standard diet, in SWR/J mice, featuring a higher metabolic health and a better triglyceride metabolism compared to common BALB/cByJ and C57BL/6J strains, a >threefold increase in hepatic basal mRNA levels of macroH2A1.2, among the top 15 upregulated genes, was found [24]. Conversely, in genetic or dietary mice models of non-alcoholic fatty liver disease (NAFLD), a disorder that is present in 90% of obese subjects, the hepatic content of macroH2A1.2, but not of macroH2A1.1, is augmented [25].

The in vivo role of macroH2A1 isoforms in lipid metabolism and obesity is thus unclear. Here, we challenged newly generated macroH2A1.2–EGFP transgenic (Tg) mice [26] with an obesogenic high fat diet (60% energy from lard): Our findings identify macroH2A1.2 as a new and potent epigenetic inhibitor of adipogenesis. Its systemic overexpression leads to a spectacular protection from obesity and its related complications. Mechanistically, macroH2A1.2 strongly impaired adipogenesis, both in vitro and in vivo.

## Results

Differential association between macroH2A1.1 and macroH2A1.2 protein levels and BMI in human adipose tissue.

**Fig. 1:**
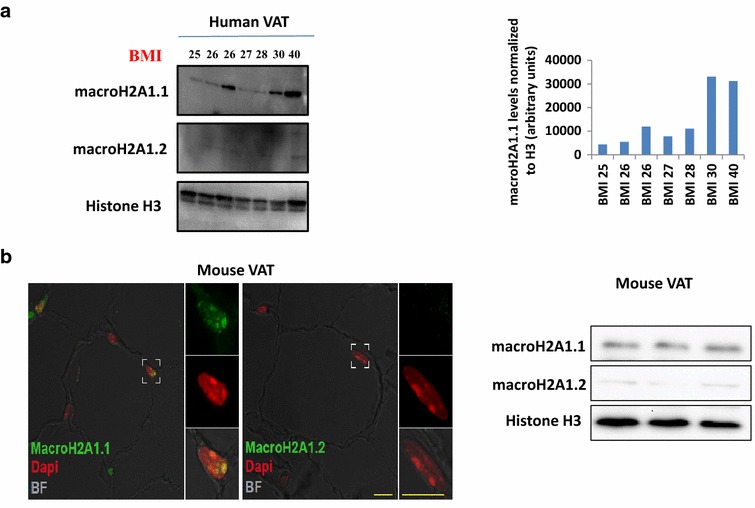
Expression of macroH2A1 isoforms in human and mouse adipose tissue. **a** *Left panel* representative immunoblots of visceral adipose tissue (VAT) human biopsies from subjects with increasing BMI (25, 26, 27, 28, 30, 40), using antibodies against macroH2A1.1, macroH2A1.2 and H3. *Right panel* densitometric quantification of macroH2A1.1 levels normalized to H3 loading control as in the *left panel*, expressed as arbitrary units. **b** *Left* representative confocal images of adipose tissue sections immunolabelled for macroH2A1.1 or macroH2A1.2. MacroH2A1.1 (*left panel*, *green*) is highly expressed in mouse adipocytes, and higher magnification inset shows its subcellular localization in macrochromatin bodies and overlapping signals with DAPI (*red*) are shown in *yellow*. MacroH2A1.2 (*right panel*, *green*) appears not expressed in mice adipose tissue. *Scale Bars* 10 μm. *Right* representative immunoblots of visceral adipose tissue from three wild-type mice, using antibodies against macroH2A1.1, macroH2A1.2 and H3

In various cell types, macroH2A1.1 displays an anti-proliferation role, while macroH2A1.2 has an anti-differentiation and pro-proliferative role [14–17, 19, 20]. Adult adipocytes are considered terminally differentiated cells. Here, we employed human visceral adipose tissue from obese subjects versus mildly overweight subjects to study the correlation between macroH2A1 isoform protein levels and body weight. Visceral adipose tissue biopsies were excised from patients with body mass index (BMI) ranging from 25 to 40, while they underwent abdominal surgery. Patient characteristics (sex, age, pathology) are described in Additional file 1: Supplementary Table I (Supplementary Material). Consistent with macroH2A1.2 being expressed at low levels in differentiated tissues, immunoblotting analysis showed that it was barely detectable at high exposure in human adipose tissue (Fig. 1a). Conversely, macroH2A1.1 isoform was found expressed, with a trend toward higher levels in subjects with high BMI (30–40) compared to subjects with lower BMI (25–26, mildly overweight) (Fig. 1a). Consistent with human data, confocal immunofluorescence analysis of white adipocytes in adipose tissue from wild-type mice revealed strong nuclear positivity (*green*) of macroH2A1.1 and absence of macroH2A1.2 isoform (Fig. 1b, left). Immunoblotting analysis confirmed a very weak expression of macroH2A1.2 compared to macroH2A1.1 in the adipose tissue (Fig. 1b, right).

### MacroH2A1.2 transgenic (Tg) mice are leaner independently of food intake and energy expenditure

**Fig. 2:**
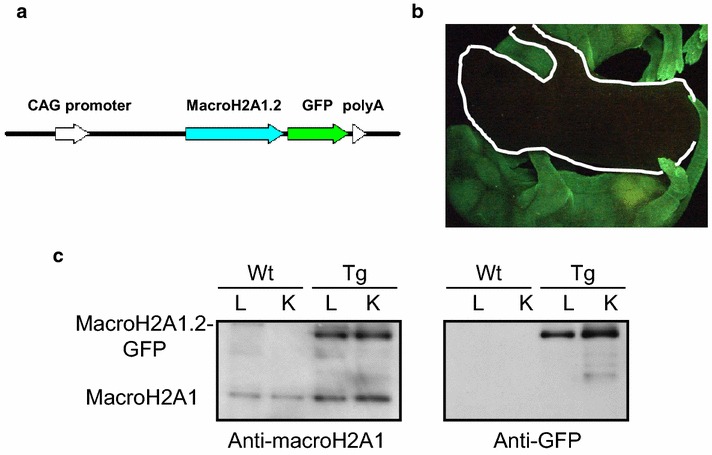
MacroH2A1.2-GFP transgenic mice (adapted from [26], licence no. 3766141157990). **a** Structure of the vector expressing macroH2A1.2–EGFP (pCXMH2A/EGFP). Mouse macroH2A1.2 tagged with EGFP was expressed under the control of the CAG promoter with a cytomegalovirus early enhancer element and a chicken b-actin promoter. **b** Stereomicroscopic analysis. The macroH2A1.2–EGFP transgenic mouse (Tg) shows green fluorescence in the skin. The *white line marks* the position of a wild-type mouse. **c** Immunoblot analysis. Nuclear extracts (10 µg/lane) of liver (L) and kidney (K) were prepared from wild-type (WT) and transgenic (Tg) female mice and analyzed using anti-GFP and anti-macroH2A1 antibodies

**Fig. 3:**
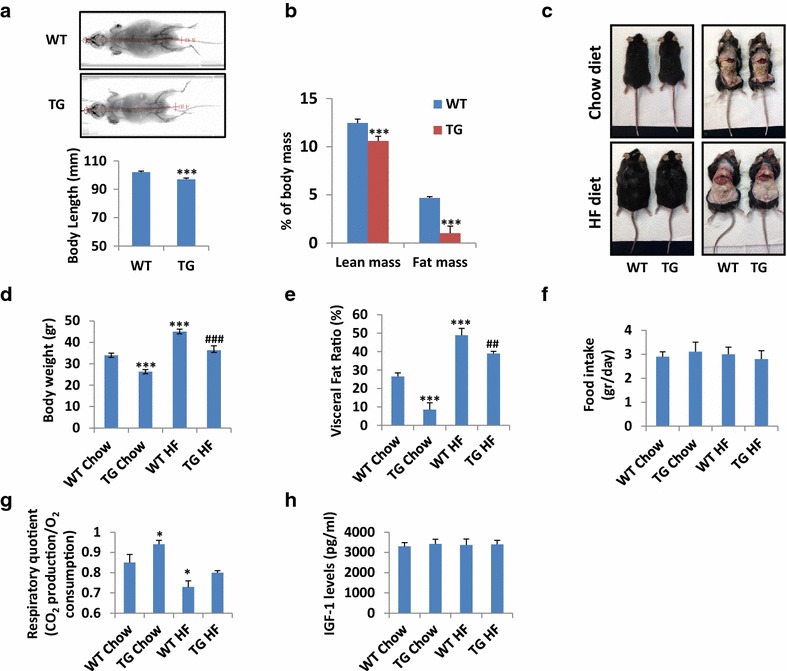
Metabolic phenotyping of macroH2A1.2 Tg mice. **a** CT scan of a representative WT and macroH2A1.2 Tg mouse (*upper panels*), image-assisted quantification of body length (*lower panel*); **b** lean and fat masses were determined by CT scan **c** representative pictures of WT and macroH2A1.2 Tg mice fed a chow diet (*upper panels*) or a HF diet (*lower panels*) mice, with a dorsal view (*left panels*) and a ventral view of peritoneal cavity showing internal organs upon killing (*right panels*); **d** body weight in WT and macroH2A1.2 Tg mice fed a chow or a HF diet at the experimental end point; **e** visceral fat ratio as determined by EchoMRI/CT scan in the four mice groups; **f** food intake in the four mice groups was assessed; **g** respiratory CO2/O2 exchange ratio as determined by metabolic cages; **h** IGF-1 blood levels, measured by ELISA (Milliplex) upon killing. Data are expressed as means} S.E.M. (n = 8–9 per group). *p < 0.05, ***p < 0.001 change versus WT fed a chow diet; ^##^ *p* < 0.01 change versus WT fed a HF diet

As human adult adipose tissue seems devoid of macroH2A1.2, we sought to study the effect or reintroducing the protein by systemic transgenic overexpression. We used a fusion plasmid (pCX-MH2A/EGFP) consisting of a CAG promoter, a chimeric cDNA encoding mouse macroH2A1.2 (GenBank accession number AF171080) and a fusion polypeptide with EGFP at the C terminus of macroH2A1.2 to generate macroH2A1.2–EGFP transgenic (Tg) mouse lines by DNA microinjection into pronuclear stage embryos [26] (Fig. 2a). Tg mice were healthy and fertile, and green fluorescence could be detected neonatally throughout the body [26] (Fig. 2b). The fusion protein could be easily detected in nuclear extracts of internal organs such as the kidney and liver by immunoblot analysis: A band of 42 kDa was detected in both wild-type and Tg mice using an anti-macroH2A1 antibody, whereas a band of 67 kDa was detected only in Tg organs, consistent with the expected size of the macroH2A1.2–EGFP and detected with an anti-GFP antibody [26] (Fig. 2c). Endogenous macroH2A1.1 expression in these organs was not changed upon overexpression of macroH2A1.2–EGFP transgene (*data not shown*). In the visceral adipose tissue, macroH2A1.2 immunopositivity was detected by confocal microscopy in the Tg, but it was absent in wild-type mice (Additional file 2: Figure S1). Quantitative EchoMRI/CT scan showed that Tg mice were in average ~5% shorter than wild type (Fig. 3a, *p* < 0.0001), with ~20% less lean mass and ~fivefold lower fat mass (Fig. 3b, *p* < 0.0001). Consistently, macroH2A1.2 Tg mice were also macroscopically leaner under a standard (chow) diet (Fig. 3c, upper panels). Similarly, when fed an obesogenic (12 weeks, 60% energy from lard [22]) high fat (HF) diet, macroH2A1.2 Tg mice appeared leaner and protected from fat induced-increased adiposity to the naked eye (Fig. 3c, lower panels). Accordingly, body weight of age-matched Tg mice was strikingly lower than wild-type mice both under a chow diet (26.4 ± 0.9 versus 33.9 ± 1.1, *p* < 0.0001) and under a HF diet (36.3 ± 2.1 vs 44.9 ± 1.2, *p* < 0.0001) (Fig. 3d). Of note, the weight of macroH2A1.2 Tg mice fed an obesogenic HF diet was not statistically different than the baseline one of wild-type mice fed a chow diet (36.3 ± 2.1 versus 33.9 ± 1.1, ns). These variations in body weight were mirrored by profound differences in the visceral adipose fat ratio as determined by EchoMRI/CT scan analysis (Fig. 3e): macroH2A1.2 Tg mice displayed more than threefold lower visceral adiposity compared to wild type fed a chow diet (8.6 ± 3.7 versus 26.5 ± 2, n = 8, *p* < 0.0001). Consistently, under HF diet Tg mice accumulated significantly less total visceral fat than wild-type mice (Fig. 3e). These large differences in body weight and adiposity could not be explained by a decrease in food intake in Tg animals, as both genotypes were found to eat the same amounts (Fig. 3f). The respiratory exchange ratio (RER) is the ratio between the amount of CO_2_ produced and O_2_ consumed by breathing. Measuring this ratio can be used for estimating which fuel (carbohydrate or fat) is being metabolized to supply the body with energy. We observed significant increase in basal RER from 0.87 ± 0.03 in wild-type mice to 0.97 ± 0.01 in macroH2A1.2 Tg mice (*p* < 0.05) (Fig. 3g), reflecting a switch from an energy consumption consisting of a mix of carbohydrates and fat to an energy consumption indicative of carbohydrate being the predominant fuel source, in Tg animals. Upon a HF diet, wild-type animals had a decreased RER compared to chow diet fed littermates (0.78 ± 0.04, *p* < 0.05) indicating fat as the predominant fuel source, while Tg mice displayed a RER, 0.84 ± 0.01, similar to baseline wild-type levels, i.e., reflecting energy consumption combining carbohydrates and fat (Fig. 3g). IGF-1 blood levels were similar in wild-type and Tg mice indicating that differences in body weight and size were independent of IGF-1 (Fig. 3h). Overall, these data demonstrate that macroH2A1.2 Tg have reduced total and visceral fat depots and an increased energy expenditure from carbohydrates.

### MacroH2A1.2 Tg mice are more glucose tolerant and insulin sensitive, and display smaller pancreatic islets

**Fig. 4:**
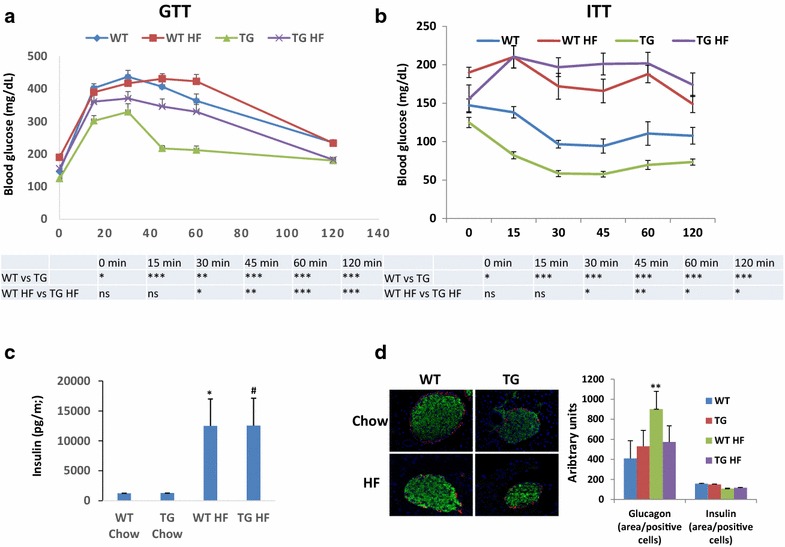
Responsiveness to glucose and insulin and pancreatic islets morphology in macroH2A1.2 Tg mice (Tg). **a**, **b** GTT and ITT were performed in WT and Tg mice fed a chow or a HFD following a 6 h fast. Mice were injected with 2 g glucose/kg of body weight intraperitoneally, and blood glucose concentrations were measured at the indicated time points. **c** Insulin blood levels, measured by ELISA (Milliplex) in the four mice groups upon killing. **d** *Left panels*: merged representative example of immunofluorescence staining for insulin (*green*), glucagon (*red*) and DAPI (4′,6-diamidino-2-phenylindole; *blue*), in control islets and macroH2A1.2 Tg mice upon HF diet; *right panel*: imaging-assisted quantification of glucagon and insulin content, expressed as the ratio area/positive cells. **c**, **d** Data are expressed as mean ± S.E.M. (n = 8–9 per group). *p < 0.05, **p < 0.01 macroH2A1.2 Tg versus WT. ^#^ *p* < 0.05 macroH2A1.2 Tg HF versus chow diet

In comparison with wild-type mice, an improvement in metabolic health is thus observed in Tg mice; we therefore also investigated the ability of Tg mice to respond to a glucose or insulin challenge. Basal glucose levels were considerably lower in Tg versus wild-type mice, fed a chow (124.9 ± 11.4 versus 147.25 ± 8.86, *p* < 0.05) or a HF diet (155.67 ± 10.6 versus 190 ± 11.3, *p* < 0.0001) (Fig. 4a, b). In glucose tolerance tests (GTT), glucose levels remained significantly lower in macroH2A1.2 Tg mice at every time point, compared to wild-type littermates, both upon a chow or a HF diet (Fig. 4a). Insulin tolerance tests (ITT) showed that the insulin-mediated decrease in glycemia was much more pronounced and statistically significant in Tg mice versus wild-type mice at every time measured (*p* < 0.0001 at 15, 30, 45, 60, 120 min time points) upon a chow diet (Fig. 4b). Upon a HF diet, statistical differences were observed between macroH2A1.2 Tg and wild-type mice only after 30 min (Fig. 4b). To gain insight into the mechanism by which systemic glucose tolerance is improved in chow and HF diet fed macroH2A1.2 Tg mice, we characterized insulin-induced AKT signaling in the skeletal muscle, liver and adipose tissues under insulin-stimulated conditions (0.75 U kg − 1 body weight, injected 15 min before killing) (Additional file 3: Figure S2). AKT phosphorylation (Ser473) was increased in insulin-responsive peripheral tissues of macroH2A1.2 Tg mice fed either a chow or a HF diet compared with wild-type controls (Additional file 3: Figure S2). Circulating insulin levels did not differ between genotypes under a chow diet, and they were found increased to the same extent upon HF diet (Fig. 4c). However, pancreatic islets were strikingly smaller in macroH2A1.2 Tg compared to wild-type mice (Fig. 4d, left panels); semi-quantitative double immunofluorescence confocal analysis in pancreatic islets showed a similar insulin content in beta cells in both genotypes, with a not significant trend toward a decreased glucagon content in alpha cells in Tg mice (Fig. 4d, right panels). Hence, macroH2A1.2 Tg mice are more glucose tolerant and insulin sensitive, and display smaller pancreatic islets.

### MacroH2A1.2 Tg mice have decreased hepatic and pancreatic fat content and inflammation upon a HF diet

**Fig. 5:**
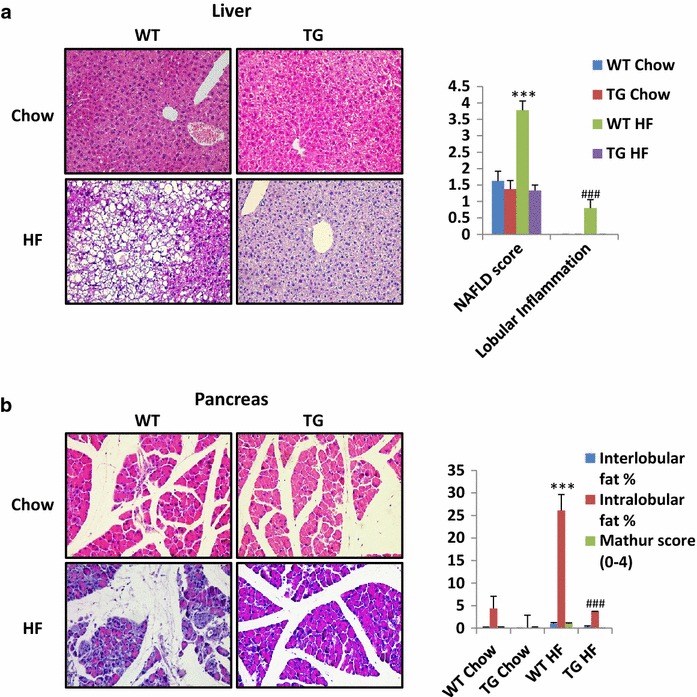
Liver and pancreas histological analyses. **a** *Left*: representative pictures from hematoxylin and eosin staining of liver sections around the lobular areas in wild-type and macroH2A1.2 Tg mice fed with a HF diet. *Right*: NAFLD and inflammation were scored using a semiquantitative system that grouped histological features into broad categories (steatosis, hepatocellular injury, portal inflammation, fibrosis and miscellaneous features) [48]. **b** *Left* representative pictures from hematoxylin and eosin staining of pancreatic sections in wild-type and macroH2A1.2 Tg mice fed with a HF diet. *Right* an evaluation of interlobular and intralobular fat % in pancreas sections was performed, and combined into a Mathur score [49]

Obesity is almost invariably associated with NAFLD and to non-alcoholic fatty pancreas disease (NAFPD), two interrelated conditions characterized by parenchymal triglyceride accumulation and inflammation. NAFLD and NAFPD are risk factors to develop cirrhosis and cancer [1, 27]. We sought to analyze the lipid content in the liver of macroH2A1.2 Tg versus wild-type mice: H&E staining revealed evident differences, with a total protection from lipid accumulation in Tg compared to wild-type animals that developed instead extensive mixed micro/macro/vesicular NAFLD upon HF diet (Fig. 5a, left panels). Accordingly, NAFLD and lobular inflammation scores were highest in livers of wild-type mice fed a HF diet; conversely, NAFLD score of Tg animals upon HF diet was similar to the one of wild-type mice fed a chow diet (Fig. 5a, right panels). H&E staining of pancreata revealed a marked decrease in fat infiltration in Tg mice versus wild-type mice (Fig. 5b, left panels). Histological analysis revealed also that upon a chow diet, wild-type mice contained ~5% of intralobular fat, while Tg mice were devoid of it; upon a HF diet, Tg mice accumulated only <5% of intralobular fat, while pancreata from wild-type mice displayed ~25% intralobular, ~1–2% interlobular fat accumulation and ~2 of Mathur score (Fig. 5b, right panels). MacroH2A1.2 Tg mice are thus protected against HF-induced NAFLD and NAFPD, consistent with their protection from obesity.

### macroH2A1.2 counteracts adipogenesis in vivo and in vitro

**Fig. 6:**
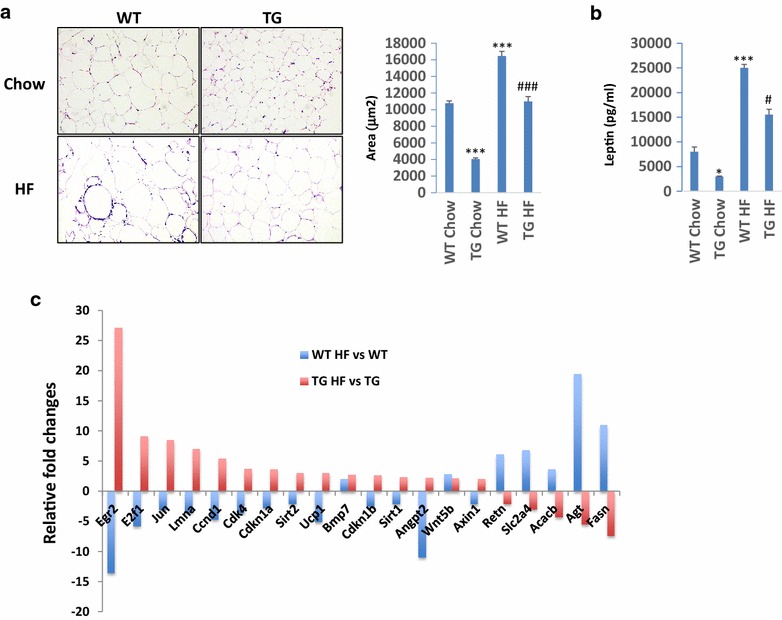
**a** *Left* representative pictures from hematoxylin and eosin staining of visceral white adipose tissue sections in wild-type (WT) and macroH2A1.2 Tg (Tg) mice fed with a HF diet. *Right* quantification of adipocyte area (μm^2^). **b** Leptin blood levels, measured by ELISA (Milliplex) in the four mice groups upon killing. Data are expressed as mean ± S.E.M. (*n* = 8–9 per group). *p < 0.05, ***p < 0.001 change versus WT fed a chow diet; ^#^ *p* < 0.01 change versus WT fed a HF diet. **c** Adipose gene expression profiling expressed in fold change and relative to HF diet compared to chow diet either in WT or in Tg mice. Results are represented as means of 3 mice per condition (WT, WT + HF diet, Tg, Tg + HF diet). All differences, with the exception of *BMP7* and *WNT5B* (not significant), are highly significant (*p* < 0.0001)

**Fig. 7:**
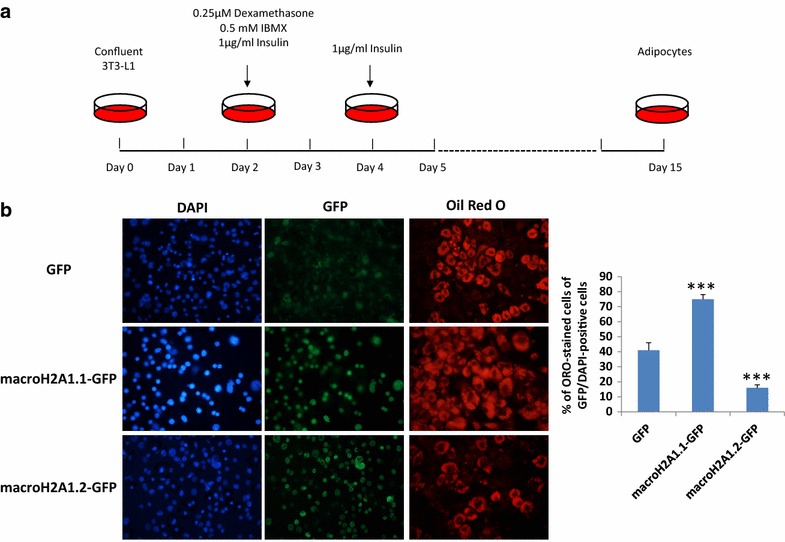
**a** Schematic protocol for 3T3-L1 differentiation from pre-adipocytes into mature adipocytes [28]. **b** *Left panels* 3T3-L1 pre-adipocytes with lentiviral-mediates stable expression of GFP, macroH2A1.1-GFP and macroH2A1.2-GFP were induced to differentiate into mature adipocytes as in (**a**). At the 15th day of differentiation, cells grown on coverslips were stained with Oil Red O (ORO) solution and counterstained with DAPI for nuclei. ORO was visualized with fluorescence microscopy. *Right panel* the percentage of ORO-stained cells was quantified over the total of GFP/DAPI-positive cells, averaging 10 different random fields in 3 separate experiments. Results are represented as mean ± S.E.M. ***p < 0.001 change versus GFP

Our data indicate that macroH2A1.2 Tg mice do not display important differences in food intake as compared to wild-type mice. We thus hypothesized that the striking protection from obesogenic diet-induced increase in body weight and adiposity in these animals could be attributed to an inhibition of adipogenesis. Adipocytes are the major storage site for fat in the form of Tgs, and this can be accomplished in two different ways, by expanding the available adipose cells or by recruiting new fat cells upon differentiation. Histological analyses of visceral white adipose tissues revealed a ~60% decrease in adipocyte size (area) in Tg mice versus wild-type mice even upon a chow diet (Fig. 6a). Upon a HF diet, Tg animals displayed adipocytes on average ~35% smaller than wild type (Fig. 6a). Variations in the circulating concentration of adipose tissue-secreted adipokine leptin in the four mice groups correlated well with the size of body fat stores (Fig. 6b). To explore the molecular mechanisms that might be involved in the decreased adiposity of macroH2A1.2 Tg mice, we used a gene array profiling the expression of 84 key genes involved in white adipose tissue adipogenesis, including hormones, adipokines, enzymes and transcription factors. Using a twofold cutoff difference in mRNA expression upon HF diet in either of the two groups of animals, we identified 20 genes, 18 of which were oppositely regulated in the adipose tissue of macroH2A1.2 Tg mice compared to wild-type mice (Fig. 6c, Additional file 1: Supplemental Table III). Pro-differentiation and pro-adipogenic genes *ACACB*, *AGT*, *FASN*, *RETN* and *SLC2A4* were significantly upregulated in wild-type adipose tissue and downregulated in macroH2A1.2 Tg adipose tissue when mice were fed a HF diet (Fig. 6c). Conversely, anti-adipogenic genes *E2F1*, *EGR2*, *JUN*, *LMNA,* anti-inflammatory genes *SIRT1*, *SIRT2,* thermogenic gene *UCP1* and proliferation-regulating genes *ANGPT2*, *CCND1*, *CDKN1A*, *CDKN1B*, were upregulated in macroH2A1.2 Tg adipose tissue and downregulated in wild-type adipose tissue when mice were fed a HF diet (Fig. 6c). *BMP7* and *WNT5B* were found two–threefold upregulated in the adipose tissue of both genotypes upon a HF diet (Fig. 6c, d). To understand whether macroH2A1.2 could affect the differentiation of pre-adipocytes into mature adipocytes, we used the well-established murine 3T3-L1 cell model. Stable expression of GFP, macroH2A1.2, and its sister splicing variant macroH2A1.1, in 3T3-L1 pre-adipocytes was achieved by lentiviral transduction as previously described [12], and differentiation into mature adipocytes was obtained through a 15 days long protocol based on the sequential addition of dexamethasone, IBMX and insulin [28] (Fig. 7a). Expression of macroH2A1.1 transgene did not have an effect on endogenous macroH2A1.1 protein levels that were stable along differentiation (day 1–5–15, Additional file 4: Figure S3). Conversely, expression of macroH2A1.1 and macroH2A1.2 transgenes led to markedly decreased levels of endogenous macroH2A1.2 at all time points of the differentiation protocol, compared to GFP-expressing control cells (Additional file 4: Figure S3). Of note, in GFP-expressing control cells macroH2A1.2 endogenous levels decreased during the differentiation of pre-adipocytes into adypocytes (day 5 and day 15 compared to day 1, Additional file 4: Figure S3), which is consistent to the low levels of macroH2A1.2 in adult human and mouse VAT (Fig. 1). At the end point of the protocol, mature 3T3-L1 adipocytes were analyzed for lipid content using Oil Red O (ORO) staining: We found that, compared to GFP-overexpressing cells, macroH2A1.2-GFP-overexpressing cells displayed a ~2.5-fold decrease in lipid content, while macroH2A1.1-overexpressing cells accumulated ~1.7-fold more lipids (Fig. 7b). MacroH2A1.2-dependent mRNA upregulation of *RETN, E2F1* and *EGR2*, together with downregulation of *FASN,* was observed in 3T3-L1 adipocytes, mirroring the in vivo data (Additional file 5: Figure S4). In vitro and in vivo data collectively suggest that macroH2A1.2 might impair adipocyte differentiation while, in the 3T3-L1 model, overexpression of macroH2A1.1 leads to augmented lipid accumulation. Interestingly, macroH2A1.1 has been consistently reported to be highly expressed and have an anti-proliferative action, while macroH2A1.2 was expressed at low levels, in differentiated cells [14]. Consistently, confocal immunofluorescence analysis of adult heart tissue, which is slowly or not proliferating and possesses scarce regenerative capacity, in WT mice fed a chow diet revealed predominant expression of macroH2A1.1 and not of macroH2A1.2 (Additional file 6: Figure S5). In contrast, under the same conditions the adult mouse liver, which is a regenerative organ mainly due to the high proliferation rate of hepatocytes, both macroH2A1.1 and macroH2A1.2 are expressed (Additional file 6: Figure S5).

### Genome occupancy of macroH2A1.2 display minor changes upon adipocyte differentiation

**Fig. 8:**
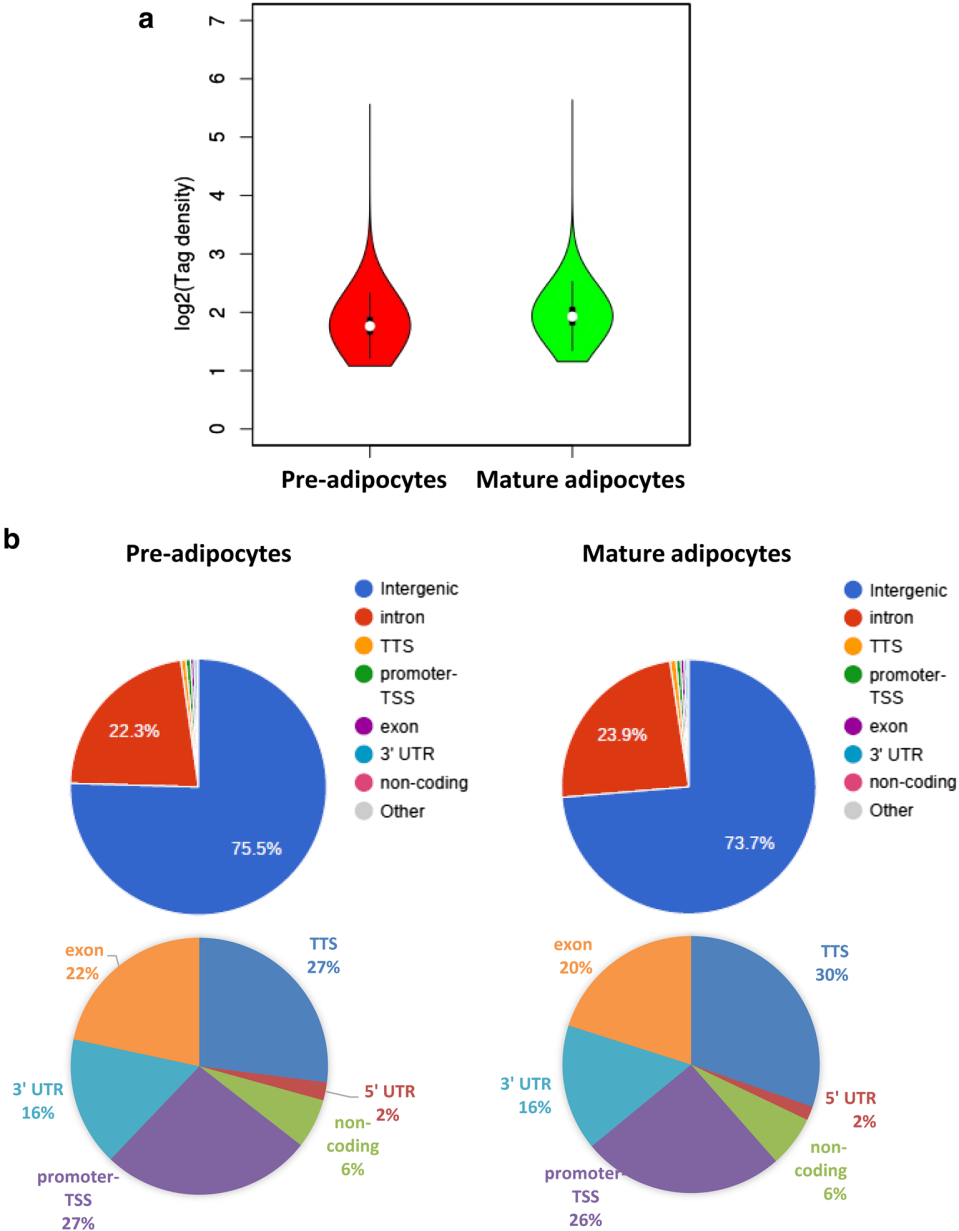
**a** Violin plots representing the density of reads pre- and post-adipogenic differentiation (pre-adipocytes and mature adipocytes, respectively), calculated with the formula: tag density = count/region length × 100 as log2 values. **b** Pie charts with annotation of the genomic regions displaying significant binding sites for macroH2A1 in pre-adipocytes and in mature adipocytes. *TTS* transcription termination site; *TSS* transcription starting site; 3′UTR 3′ untraslated region. Most of the macroH2A1.2 occupancy is intergenic or in introns (upper pies): lower pies include only the unrepresented genomic binding patterns of macroH2A1.2 (TTS, promoter-TSS, 5′UTR, 3′UTR, noncoding)

We sought to analyze if changes in chromatin occupancy macroH2A1.2 might play a role in transcriptional changes associated with adipocyte differentiation in vitro. We used ChIP-Seq, using a ChIP grade anti-GFP antibody, to isolate and sequence genomic regions associated with macroH2A1.2-GFP chimeric protein. No antibody was used as a negative control to subtract aspecific peak distribution. The data discussed in this publication have been deposited in NCBI’s Gene Expression Omnibus and are accessible through GEO Series accession number GSE85796 (https://www.ncbi.nlm.nih.gov/geo/query/acc.cgi?acc=GSE85796). Both pre- and post-3T3-L1 adipocyte differentiation, macroH2A1.2-associated reads displayed a bell shape distribution with a peak at about −7000–8000 bp upstream of TSS (Additional file 7: Figure S6). Similar binding affinity of macroH2A1.2 histone was observed in mature adipocytes and in pre-adipocytes, as determined by sequence read tag density (Fig. 8).

**Fig. 9:**
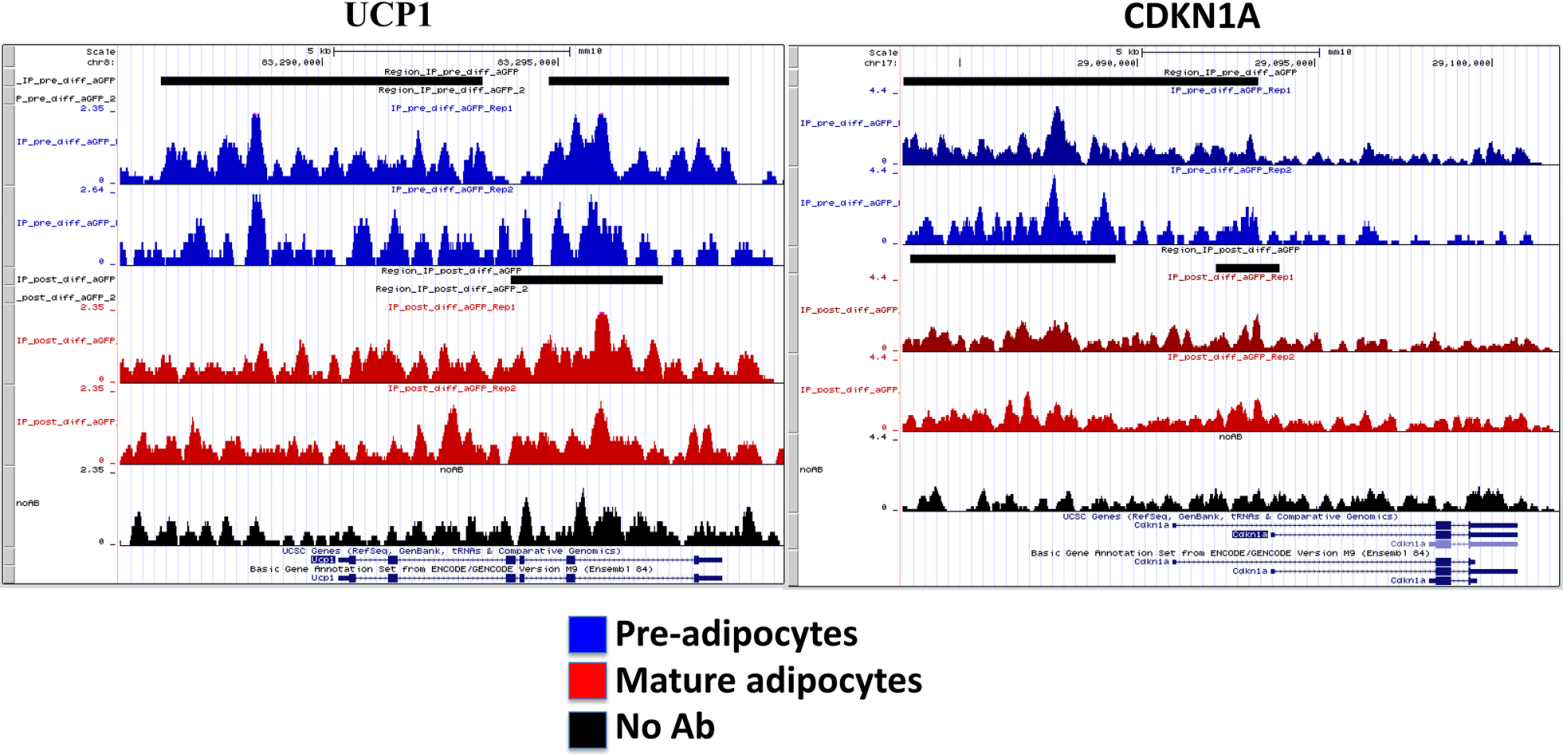
Genome browser (University of California Santa Cruz, UCSC) graphic representation of UCP1 and CDKN1A genes. Bigwigs in *blue* and *red* indicate peak distributions—reflecting macroH2A1.2 binding—in 3T3-L1 pre-adipocytes and in 3T3-L1 mature adipocytes, respectively, in biological duplicates. Bigwigs in *black* indicate aspecific peak distribution [no antibody (Ab), negative control]. *Black bars* indicate enriched regions (binding sites) with a false discovery rate (FDR) <0.01

**Fig. 10:**
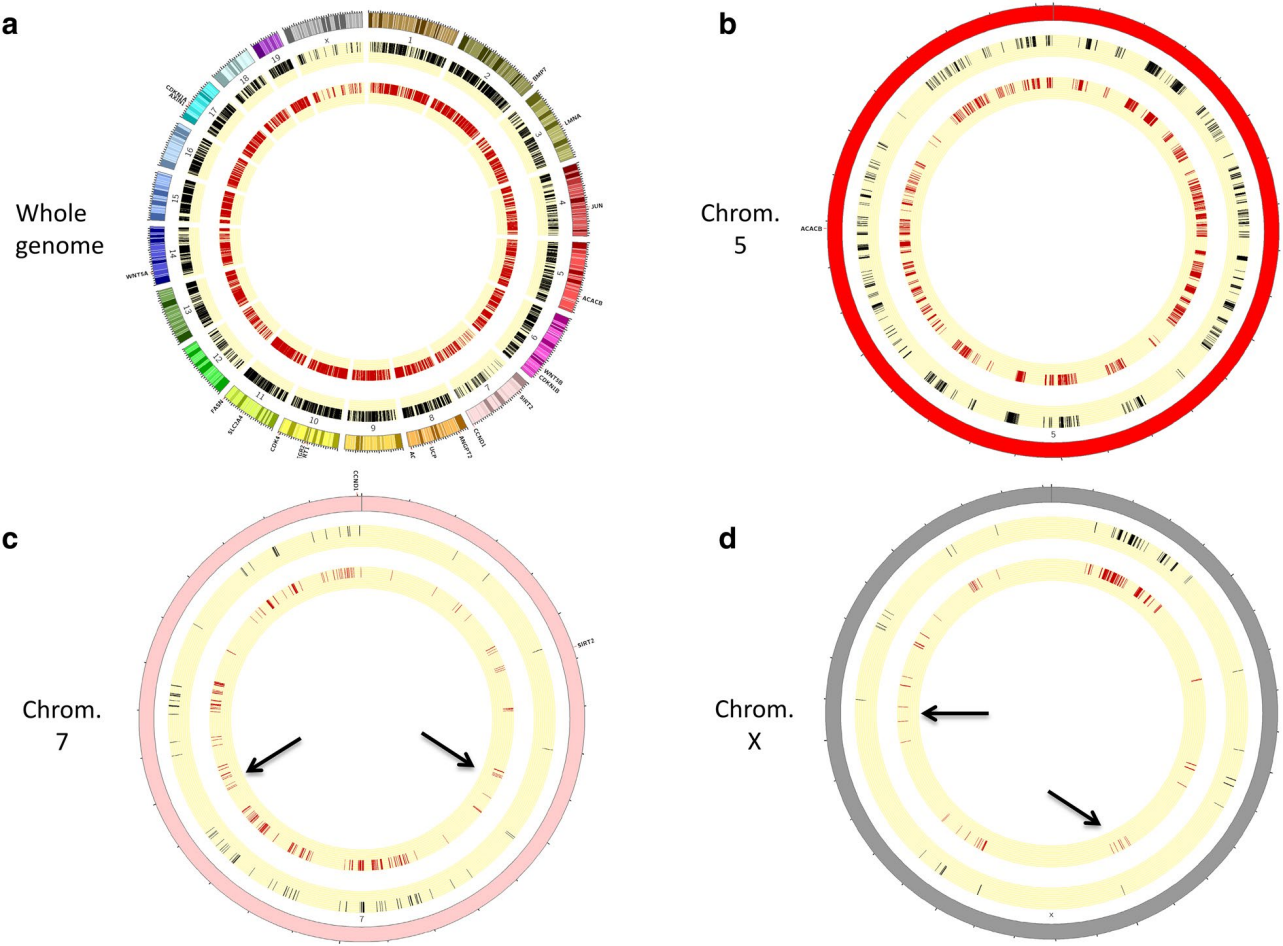
Representation of commonly and differentially bound genes in ChIP-Seq by Circos plots. **a** Circos plot of the whole 3T3-L1 cells genome, representing in the inner circle (in *red*) macroH2A1.2 significant binding regions in mature adipocytes, in the middle circle (in *black*) macroH2A1.2 significant binding regions in pre-adipocytes are represented and in the *outer circle* all chromosomes together with the localization of the 20 genes analyzed in Fig. 6. **b**, **c** and **d** represent magnifications of chromosomes 5, 7 and X, respectively, as in (**a**). *Black arrows* point to chromosomic regions differentially bound by macroH2A1.2

Binding sites were subsequently grouped by gene section, i.e., 3′ or 5′ untranslated region (UTR), coding sequence, intergenic, intron, TTS, non-coding and promoter-TSS (Fig. 8b). The frequency of occupancy showed that macroH2A1.2 binding was enriched in intergenic and intron regions, with no significant differences between pre- and post-differentiation conditions (Fig. 8b, upper panels). Similarly, filtering binding sites to exclude intergenic and intron regions, in order to highlight exons, TTS, 5′UTR, noncoding, promoter-TSS and 3′ UTR, did not evidence differences in binding patterns (Fig. 8b, lower panels). Analysis of genome occupancy of the pro- and anti-adipogenic genes (*ACACB*, *AGT*, *FASN*, *RETN, SLC2A4*, *E2F1*, *EGR2*, *JUN*, *LMNA, SIRT1*, *SIRT2, UCP1*, *ANGPT2*, *CCND1*, *CDKN1A* and *CDKN1B)* under the control of macroH2A1.2 in vivo (Fig. 6c) identified enriched regions (binding sites) with a false discovery rate (FDR) <0.01 only in 4 genes (UCP1, CDKN1A, FASN, Slc2a4), which were though inconsistent between biological duplicates and were not significantly different between 3T3-L1 pre-adipocytes and in 3T3-L1 mature adipocytes (Fig. 9 and *data not shown*). To gain a more general view, macroH2A1.2-bound genes in 3T3-L1 cells in ChIP-Seq were represented by Circos plots (Fig. 10a). Zooming to represent magnifications of example chromosomes 5, 7 and X uncovered that with the exception of few distinct regions macroH2A1.2 significant genomic binding patterns in pre-adipocytes and in mature adipocytes were very similar (Fig. 10b–d). Our genomic analysis thus showed that the process of differentiation induces modest changes in macroH2A1.2 genome occupancy that may not account for its transcriptional effects in fat cells.

**Fig. 11:**
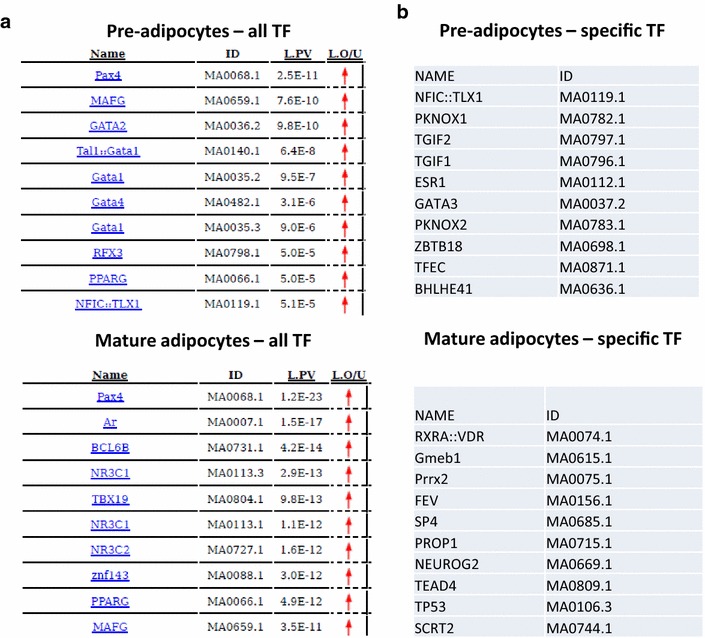
**a** Tables representing the enrichment for binding sites of known transcription factors (TF) in macroH2A1.2-binding regions in pre-adipocytes (*left*) and mature adipocytes (*right*). In each table, the *left column* contains gene names, the *middle column* indicates the matrixes generated through PscanChIP and the *right column* contains the *p* value. **b** Tables illustrating the TF-binding sites, best hits among the first one hundred, not in common, e.g., specifically enriched in pre-adipocytes or in mature adipocytes

We then hypothesized that macroH2A1.2 could modulate transcription through the recruitment/modulation of the activity of transcription factors (TF). We thus examined the binding region sequences within the two datasets (3T3-L1 pre- and post-differentiation) to search overrepresented binding motifs for TF that might play a role in mediating macroH2A1.2-dependent effects. To this aim, we used PscanChIP tool that, starting from a collection of genomic regions, evaluates both motif enrichment and positional bias within them. Interestingly, in both pre-adipocytes and in mature adipocytes paired box 4 (PAX4) ranked as the most enriched TF presenting a binding matrix among macroH2A1.2-binding regions (Fig. 11a). GATA-binding sites were overrepresented in pre-adipocytes (Fig. 11a). In addition to the common matrices enriched in both pre- and post-differentiation adipocytes (including the master TF regulator of adipogenesis PPARγ), we observed several matrices specifically overrepresented in either pre-adipocytes (NFIC:TLX1, PKNOX1, TGIF2, TGIF1, ESR1, GATA3, PKNOX2, ZBTB18, TFEC and BHLHE41) or mature adipocytes (RXRA::VDR, Gmeb1, Prrx2, FEV, SP4, PROP1, NEUROG2, TEAD4, TP53 and SCRT2) (Fig. 11b). Of note, anti-adipogenic RXRA::VDR and TP53 were overrepresented specifically in mature macroH2A1.2-overexpressing adipocytes. Several of the TFs emerging from the analysis are known to bind to homeobox genes (NFIC:TLX1, PKNOX1, TGIF1, TGIF2, PKNOX2 in pre-adipocytes; Prrx2 and PROP1 in mature adipocytes) (Fig. 11b). Other binding matrices identified TF that function in neural/muscular/bone developmental programs (ZBTB18, TFEC, BHLHE41 in pre-adipocytes, and GMEB1, SP4, NEUROG2, TEAD4 and SCRT2 in mature adipocytes), likely associating to transcriptional repression neighboring macroH2A1.2 binding in adipose cells (Fig. 11b). These data demonstrate that, despite minor changes in genomic occupancy, macroH2A1.2 might associate with different TF-binding sites upon adipocyte differentiation, suggesting a potential transcriptional mechanism.

## Discussion

Obesity and obesity-related disorders are attaining pandemic proportions with more than a third of the population living in Western countries, with profound repercussions on the health systems. Epigenetic mechanisms are key players in the pathophysiology of obesity and related complications. Among these mechanisms, canonical histones can be replaced with histone variants that alter chromatin structure and DNA accessibility.

MacroH2A1 is a histone variant of histone H2A present in two spicing isoforms, macroH2A1.1 and macroH2A1.2, playing diverse roles in cell differentiation and plasticity [8–11, 14–18]. Previous conflicting evidence showed that histone variant macroH2A1 whole gene transcriptional activity either favors lipid accumulation [22] or it may be protective against it [21, 23], in either cases with a mild phenotype. This is the first study showing that macroH2A1.2 isoform strongly protects against HF-induced obesity. Mice bearing a chimeric macroH2A1.2-GFP transgene displayed drastically reduced total and visceral adiposity compared to their wild-type littermates both upon a chow or a HF diet. Obesity-induced liver and pancreatic damages in terms of fat accumulation and inflammation were completely wiped out by macroH2A1.2 transgene in mice which looked healthy as the wild type fed a chow diet, without differences in food intake. Tg mice typically relied on a mix carbohydrate/fat burn for energy consumption, while wild-type animals relied rather on fat consumption, which could be explained by the very low levels of basal body adiposity in Tg mice. MacroH2A1.2 Tg mice were also slightly shorter than controls; however, we did not find differences in IGF-1 levels, which are implicated in growth and adiposity extent in mice [29]. Tg mice were also, to a large extent, more insulin sensitive and glucose tolerant than wild-type mice. The reduced size of pancreatic islets in Tg mice is reminiscent of a caloric restriction regimen [30]. Glucagon levels are often disrupted in obese individuals: accordingly, the number of glucagon-producing α-cells was perturbed in wild-type but not in Tg mice when fed a HF diet. Altogether, these strong evidences of metabolic health unrelated to alterations of food intake or GH/IGF-1 axis led us to hypothesize that the leanness of the Tg mice might be intrinsic to the adipose tissue and due to reduced adipogenesis at the molecular level. Reduced adipogenesis could translate into less circulating fat and triglycerides that might damage other peripheral organs such as the liver and the pancreas, without directly impinging on energy expenditure and hunger. Expression analysis of mRNA transcripts implicated in adipogenesis uncovered downregulation of pro-adipogenic genes *ACACB*, *AGT*, *FASN*, *RETN* and *SLC2A4* and upregulation of anti-adipogenic genes *E2F1*, *EGR2*, *JUN*, *LMNA,* anti-inflammatory genes *SIRT1*, *SIRT2,* thermogenic gene *UCP1* and pro-proliferative genes *ANGPT2*, *CCND1*, *CDKN1A*, *CDKN1B* in macroH2A1.2 Tg mice, which is consistent with a reduction of adipocyte mass and with a potential expansion of the undifferentiated pre-adipocyte cellular pool. Consistently, macroH2A1.2-GFP-overexpressing 3T3-L1 pre-adipocytes showed strongly reduced lipid content upon differentiation into adipocytes, as observed in Tg mice. It is has been shown that 3T3-L1 cell differentiation associates with genome-wide epigenetic dynamic changes in the DNA demethylation/methylation ratio in a time- and stage-dependent manner [31]; DNA hypomethylating agent decitabine blocked the 3T3-L1 adipogenic process [31]. In the context of cancer cells, macroH2A1 incorporation antagonizes the anti-proliferative effects of decitabine [12, 13]. We postulate that macroH2A1.2 might interfere with DNA methylation events during the adipogenic gene expression program.

Surprisingly, stable overexpression of macroH2A1.1, the second splicing variant of macroH2A1 gene, yielded opposite effects, with large lipid accumulation upon differentiation. Our data in mice and humans suggest also that adipose tissue display endogenous low or absent expression of macroH2A1.2, while macroH2A1.1 is present, and its levels increase upon a HF diet-induced obesity in mice, and in obese compared to mildly overweight individuals; however, these patients were enrolled for other pathologies and this could have an impact on the expression of macroH2A1 isoforms. Since depletion of the whole macroH2A1 gene [22] has also, although milder, anti-adipogenic effects in mice fed a HF diet, we argue that macroH2A1.1 has a stronger pro-adipogenic role than the protective one of macroH2A1.2. Generation of macroH2A1.1 transgenic murine models will prove its mechanistic role and tissue-specific interaction with macroH2A1.2 during the development of obesity in vivo. Genome-wide distribution of macroH2A1 histone variants in mouse liver chromatin indicate that macroH2A1 functions primarily as a repressor in adult liver [32].

Variations in macroH2A1 transcriptional activities to a large extent independent of genome occupancy in response to nutritional and DNA methylation status have been reported in cancer cells [9, 11, 12]. Consistent with these studies, our ChIP-Seq analyses comparing macroH2A1.2 Tg genome binding between 3T3-L1 pre-adipocytes and 3T3 post-differentiated adipocytes revealed high degree of similarity in genome binding patterns, including within the bodies of the subset of genes involved in the adipogenic process that we analyzed. Variations in the expression and sequential binding patterns of key TF (GATA, C/EBPβ and −δ (C/EBPδ), PPARγ and C/EBPα) function in the adipogenic differentiation program. Our in silico analysis of TF-binding sites in proximity of macroH2A1.2 genome binding revealed that GATA-binding sites were overrepresented in the proximity of macroH2A1.2 binding in 3T3-L1 pre-adipocytes compared to differentiated cells, consistent with their role in adipocyte precursors and with their downregulation setting the stage for terminal differentiation. In mature adipocytes, RXRA::VDR and TP53-binding sites were enriched, which might reflect their anti-adipogenic transcriptional effects [33, 34]. The enrichment of consensus sequences for TF controlling homeobox genes, including the overrepresented PAX4, in macroH2A1.2 associates chromatin reads in the context of adipocyte differentiation deserves further investigation. In addition to potential dynamic interactions with TFs, it is unclear how posttranscriptional modifications might affect histone variants function without changes in occupancy. Adipose tissue is at the nexus of processes involved in health span and metabolic dysfunction: Progression of age-related fat tissue dysfunction follows different trajectories across different fat depots, with fat becoming redistributed from subcutaneous to intraperitoneal depots and ultimately ectopic sites. This is associated with metabolic disturbances. The pre-adipocytes from which new fat cells develop switch into a pro-inflammatory, tissue-remodeling state in old age, instead of differentiating into fat cells [35]. In cellular senescence, proliferation becomes arrested and cells acquire a pro-inflammatory senescent secretory phenotype (SASP), with release of chemokines and cytokines [35]. We and others have shown that macroH2A1.1 ectopic expression sustains SASP in fibroblasts and hepatoma cells [12, 36]; a similar signaling loop might take place in the adipose tissue. How to boost macroH2A1.2 expression/activity, to the detriment of macroH2A1.1, with an anti-obesity therapeutic purpose? Some of the switch factors controlling macroH2A1 splicing into macroH2A1.1 or macroH2A1.2 isoforms have been identified in cancer cells: QKI and RNA helicases Ddx17/Ddx5 [37, 38]. Epigenetic splicing regulatory strategies are already being explored for cancer therapy and might hold also a potential against obesity and its related complications.

## Conclusions

Histone variant macroH2A1.2 is an unprecedented chromatin component that, if overexpressed, inhibits pro-adipogenic transcriptional programs, fat deposition and massively protects mice from high fat diet-induced obesity. Given that this histone is not expressed in human differentiated adipose tissue, it is possible to envisage that strategies aiming at its reintroduction in fat tissues could pave the way for new anti-obesity therapies.

## Methods

### Human biopsies

Seven patients with BMI from 25 to 40 were enrolled (Additional file 1: Supplementary Table I, Supplementary Material). Patient underwent surgical procedures (Whipple’s, right hepatectomy, wedge resections of the liver) according to conditions including pancreatic adenocarcinoma and colorectal liver metastases. Informed consent was obtained from each patient. Procedures to extract adipose tissue biopsies were performed through incision that depended on the type of operation, transverse abdominal for pancreas, midline or inverted L shape for liver. The incision of the skin was performed with the blade knife until the subcutaneous tissue was visualized. Around the umbilicus, a small part of the subcutaneous fat (0.5 cm^3^) was resected with the scissors. Specimens were frozen at −80 °C before further characterization by immunoblotting. The study was approved by the UCL Royal Free Biobank Ethical Review Committee (NRES Rec Reference: 11/WA/0077). Tissues were processed in accordance with the UCL Royal Free Biobank protocols under the Research Tissue Bank Human Tissue Act licence, prior to use in research [39].

### Animals

The MacroH2A1.2–EGFP transgenic mouse line established by Soma et al. [26] was backcrossed to C57BL/6 J for 5–6 generations. Tg mice positive for the transgene were identified by PCR amplification from tail tissue genomic DNA, using the primers 5′-TGACAGAAAGCTGAAATCCATCGC-3′ and 5′-TCCAGCAGGACCATGTGATCGC-3′, and by observing whole-body EGFP fluorescence using a UVL-56 handheld UV lamp (UVP, Cambridge, UK or Upland, CA, USA). All experiments were approved by Tottori University Ethical Committee, were performed using heterozygous transgenic mice and were carried out according to the Guide for the Care and Use of Laboratory Animals of Tottori University. Six-week old wild-type (WT) and transgenic (Tg) mice were assigned into 4 groups (8–9 mice/group): WT fed with chow diet, Tg fed with chow diet, WT fed with high fat (HF) diet and Tg fed with HF diet for 12 weeks. Obesogenic diet consist 60% energy from lard [22].

### EchoMRI quantitative magnetic resonance and CT scan

EchoMRI™ quantitative magnetic resonance (QMR) technology was used to measure the body composition of live mice in terms of whole-body fat, and lean masses, according to manufacturer’s instructions: Measurements were made by placing live free moving mice into a thin wall plastic cylinder (4.7 cm, inside diameter; 0.15 cm thick) with freedom to turn about but limited to ~4 cm vertical movements by a plastic insert. After 2 min, when the measurement was completed, conscious mice returned to their home cage. Alternatively, CT scanning was performed in isofluorane-anesthetized animals at 2-mm intervals from head to tail to determine body length, or from the diaphragm to the bottom of the abdominal cavity to determine visceral fat and liver fat content, using a LaTheta™ LCT 200 in vivo micro-CT scanner, according to manufacturer’s instructions (Hitachi, Aloka Medical, Japan).

### Metabolic cages, glucose tolerance test (GTT) and insulin tolerance test (ITT)

In order to monitor in real time the metabolic gas exchange, groups of 8–9 mice per genotype on HFD were placed in indirect calorimetric cages where energy expenditure, food intake and activity were evaluated [22]: The whole-body energy metabolism in mice was calculated in vivo as previously described [22]. GTT and ITT were performed as previously described [40].

### Histology and immunofluorescence

Histological analyses and immunofluorescence staining are described in detail in the Additional file 1: Supplementary Materials and methods.

### Quantification of circulating cytokines

Insulin, leptin and IGF-1 levels were assessed in the sera of wild-type and macroH2A1.2 Tg animals, using a customized mouse MILLIPLEX^®^ MAP (multi-analyte panels) Luminex system (Merck Millipore) [22], according to the manufacturer’s instructions.

### Gene expression

A commercially available adipogenesis array (mouse RT-Profiler array, Qiagen, Italy) was used to measure genes involved in adipogenesis by qRT‐PCR in mice visceral adipose tissue; expression data were normalized to the geometric mean of three house keeping genes (Actb, GAPDH and GusB). Briefly, after homogenization, tissue RNAs were isolated using Trizol (1 ml per 100 mg), according to manufacturer’s protocol. RNA samples were purified using mini spin columns (Qiagen), quantified using Nanodrop Spectrophotometer (Thermo scientific, UK). As for 3T3-L1 cell differentiation experiments, the following QuantiTect primers (Qiagen) were used: FASN (QT00149240), RETN (QT00093450), EGR2 (QT00160125) and E2F1 (QT01079106). qRT-PCR for determining gene expression in 3T3-L1 cells was performed on 50 ng of purified RNA using the one-step QuantiFast SYBR Green RT-PCR kit (Qiagen) and the Mouse SYBR Green QuantiTect primer assay. All reactions were set up in 96-well plates using a 7700HT Real-Time PCR System (Applied Biosystems, Foster City, CA).

### Western blot

Total and cytoplasmic/nuclear/histone proteins extraction and immunoblotting analyses were performed as previously described [12]. Primary antibodies were as follows: anti-GFP (Abcam, ab13970) anti-macroH2A1.1 (Cell Signaling, Cat 12455), anti-macroH2A1.2 (Cell Signaling, Cat. 4827), anti-AKT (Cell Signaling, Cat. 9272), anti-phosphoAKT-Ser473 (Cell Signaling, Cat. 9271), anti-β-actin (Cell Signaling, Cat. 4967) and anti-H3 (Cell Signaling, Cat. 9715).

### Generation of stable clones of 3T3-L1 cells: differentiation and lipid staining

Stable expression of macroH2A1.2 variant in 3T3-L1 pre-adipocytes was achieved by lentiviral transduction as previously described [12]. Stable 3T3-L1 pre-adipocytes were cultured to differentiate into mature adipocytes according to an established 15-day protocol [28]. 3T3-L1 cells at the 15th day of differentiation seeded on coverslips were washed with PBS and fixed with 4% paraformaldehyde for 10 min at room temperature. After fixation and further washings with PBS, cells were stained with an Oil Red O solution in 40% isopropanol. Coverslips were then mounted on microscope slides with Vectashield mounting medium with DAPI, and images were collected using a Nikon Eclipse E600 microscope.

### Chromatin immunoprecipitation, sequencing and data analysis

Chromatin immunoprecipitation was performed in mice adipose tissue with a modified protocol as described previously [12, 41], using an anti-GFP antibody (ab290, Abcam). 10 ng of purified ChIP DNA was used as starting material for sequencing libraries preparation. Indexed libraries were prepared with TruSeq ChIP Sample Prep Kit (Illumina Inc.). Size distribution of each ChIP library sample was assessed by running a 1 µl aliquot on Agilent High Sensitivity DNA chip using an Agilent Technologies 2100 Bioanalyzer (Agilent Technologies). The concentration of each sample was determined by using a Qubit Fluorometer (Life Technologies). Libraries were sequenced (single read, 1 × 50 cycles) at a concentration of 10 pM/lane on HiSeq 2500 (Illumina Inc.). The raw sequence files generated (fastq) underwent quality control analysis using FASTQC (http://www.bioinformatics.babraham.ac.uk/projects/fastqc/). Reads were aligned to the mus musculus genome (assembly mm10) using Bowtie software, allowing up to 2 mismatch and considering uniquely mappable reads. Duplicate sequences were removed before peaks enrichment calculation using MarkDuplicates (Picard Tools; https://broadinstitute.github.io/picard/). Violin plots representing the density of reads were generated with the vioplot package [42]. The enriched ChIP-Seq regions were identified using Spatial Clustering for Identification of ChIP-Enriched Regions (SICER) [43] setting standard parameters and a false discovery rate of 1%. For each analyzed condition, only regions in common between the two biological replicates were considered for further analysis. The annotation of peaks to the nearest gene was performed using the annotatePeaks.pl function from HOMER [44]. Overrepresented transcription factor binding sites analysis was performed using PscanChip [45], while annotation plots were generated using ChIPseek [46]. Circos plot was generated with Circos [47].

### Statistical tests

Results are expressed as mean ± S.E.M. Comparisons between groups were performed with the parametric Student’s *t* test or the nonparametric Mann–Whitney U test, as appropriate, using GraphPad Prism software (version 5.00 for Windows, San Diego, CA, USA): A *P* value ≤0.05 was considered significant.

## Additional files

**Additional file 1.**

**Additional file 2. Figure S1.** Representative images of wild-type and macroH2A1.2 transgenic (Tg) mice adipose tissue (VAT) sections immunostained for macroH2A1.2 (*red*). Nuclei were counterstained with Hoechst (*blue*), while perilipin was immunostained to define adipose cell membranes (*green*). macroH2A1.2 expression was detected only in the VAT of Tg animals but not in wild-type animals (white arrows).

**Additional file 3. Figure S2.** Increased glucose clearance because of enhanced insulin sensitivity in the muscle, liver and adipose tissue. Mice fed a chow diet were injected with insulin (INS, 0.75 U kg − 1) 15 min before being killed, after which phosphorylation status of AKT (Ser473) was determined by western blot. Representative immunoblots are shown in the skeletal muscle, liver and adipose tissue. Immunoblots were quantified by densitometry and normalized against total protein levels of AKT. *P < 0.05, ***P < 0.001 change WT + INS vs WT; ###P < 0.001 change Tg + INS vs Tg; $P < 0.05 Tg + INS vs WT + INS.

**Additional file 4. Figure S3.** MacroH2A1.1 and macroH2A1.2 expression in 3T3-L1 pre-adipocytes and adipocytes. 3T3-L1 pre-adipocytes with lentiviral-mediates stable expression of GFP, macroH2A1.1-GFP and macroH2A1.2-GFP were induced to differentiate into mature adipocytes as in Fig. 6. At the 1st, 5th and 15th day of differentiation, histones were extracted and processed for immunoblotting with anti-macroH2A1.1, macroH2A1.2 and anti-H3-specific antibodies. Representative blots are shown, together with MW ladder.

**Additional file 5. Figure S4.** Gene expression in 3T3-L1 adipocytes. 3T3-L1 pre-adipocytes with lentiviral-mediates stable expression of GFP, macroH2A1.1-GFP and macroH2A1.2-GFP were induced to differentiate into mature adipocytes as in Fig. 6. At the 15th day of differentiation, RNA was extracted and processed for qPCR analyses with specific primers. Results were normalized to pre-differentiation gene levels. Values are represented as means (N = 3) ± S.E.M. *P < 0.05; ***P < 0.0001 change vs GFP.

**Additional file 6. Figure S5.** Representative images of liver (left panels) and heart (right panels) sections immunostained for macroH2A1.1 or for macroH2A1.2 (*green*). Both isoforms appear to be highly expressed in hepatocytes, whereas there a strong reduction in expression pattern of macroH2A1.2 is observed in mouse heart tissue. Nuclei were counterstained with DAPI.

**Additional file 7. Figure S6.** Histogram representing the distance and the frequency of macroH2A1.2-binding regions from transcriptional starting site (TSS), genome-wide.

## Abbreviations

BMI: body mass index
ChIP: chromatin immunoprecipitation
CT: computer tomography
EchoMRI: quantitative echography and magnetic resonance imaging
GTT: glucose tolerance test
ITT: insulin tolerance test
KO: knock out
ORO: Oil Red O
NAFLD: non-alcoholic fatty liver disease
NAFPD: non-alcoholic fatty pancreas disease
Tg: transgenic
