# Supplementary material for: Histone macroH2A1.2 promotes metabolic health and leanness by inhibiting adipogenesis
Source: Epigenetics Chromatin. 2016 Oct 25;9:45. doi: 10.1186/s13072-016-0098-9 (PMC5078890; doi:10.1186/s13072-016-0098-9)
Supplement: Supplementary file 1 — Additional file 1. [file 13072_2016_98_MOESM1_ESM.doc]

**Figure Legends**

**Figure S1.** Representative images of wild type and macroH2A1.2 transgenic (TG) mice adipose tissue (VAT) sections immunostained for macroH2A1.2 (*red*). Nuclei were counterstained with Hoechst (*blue*), while perilipin was immunostain to define adipose cell membranes (*green*). macroH2A1.2 expression was detected only in the VAT of TG animals but not in wild type animals (white arrows).

**Figure S2.** Increased glucose clearance because of enhanced insulin sensitivity in the muscle, liver and adipose tissue. Mice fed a chow diet were injected with insulin (INS, 0.75 U kg−1) 15 min before being killed, after which phosphorylation status of AKT (Ser473) was determined by western blot. Representative immunoblots are shown in the skeletal muscle, liver and adipose tissue. Immunoblots were quantified by densitometry and normalized against total protein levels of AKT. *P<0.05, ***P<0.001 change WT+INS vs WT; ###P<0.001 change TG+INS vs TG; $P<0.05 TG+INS vs WT+INS.

**Figure S3.** MacroH2A1.1 and macroH2A1.2 expression in 3T3-L1 pre-adipocytes and adipocytes. 3T3-L1 pre-adipocytes with lentiviral-mediates stable expression of GFP, macroH2A1.1-GFP and macroH2A1.2-GFP were induced to differentiate into mature adipocytes as in Figure 6. At the 1^st^, 5^th^ and 15^th^ day of differentiation histones were extracted and processed for immunoblotting with anti-macroH2A1.1, macroH2A1.2 and anti-H3 specific antibodies. Representative blots are shown, together with MW ladder.

**Figure S4.** Gene expression in 3T3-L1 adipocytes. 3T3-L1 pre-adipocytes with lentiviral-mediates stable expression of GFP, macroH2A1.1-GFP and macroH2A1.2-GFP were induced to differentiate into mature adipocytes as in Figure 6. At the 15th day of differentiation RNA was extracted and processed for qPCR analyses with specific primers. Results were normalized to pre-differentiation gene levels. Values are represented as means (N=3) ± S.E.M. *P<0.05; ***P<0.0001 change vs GFP.

**Figure S5.** Representative images of liver (left panels) and heart (right panels) sections immunostained for macroH2A1.1 or for macroH2A1.2 (*green*). Both isoforms appear to be highly expressed in hepatocytes whereas there a strong reduction in expression pattern of macroH2A1.2 is observed in mouse heart tissue. Nuclei were counterstained with DAPI.

**Figure S6.** Histogram representing the distance and the frequency of macroH2A1.2 binding regions from transcriptional starting site (TSS), genome-wide.

**Material and Methods**

*Histology analyses*

Parafﬁn-embedded sections of the liver, pancreas adipose tissue (4μm) were processed by haematoxylin and eosin for histological evaluation, as described previously [1, 2]. Diagnostic classiﬁcation of NAFLD was performed by applying a semiquantitative scoring system that grouped histological features into broad categories (steatosis, hepatocellular injury, portal inﬂammation, ﬁbrosis and miscellaneous features) [3]. Adipocyte area and perimeter were evaluated using the ImagePro Plus software (Media Cybernetics, Inc., Bethesda, MD, USA). An evaluation of interlobular and intralobular fat was performed on pancreas sections using the Mathur’s score [4].

*Immunofluorescence*

For double immunofluorescence of pancreatic islets, sections of pancreatic tissue were dewaxed in xylene, rehydrated in ethanol, washed in phosphate-buffered solution (PBS), incubated with unmasking solution (tri-sodium citrate 10 mM, 0.05% Tween 20) for 10 min at 60 °C and treated with blocking solution (3 % albumin bovine serum in PBS) for 30 min. Then, the sections were incubated with the first primary antibody, rabbit anti-insulin (Insulin H-86, code sc-9168, Santa Cruz Biotechnology, Europe) diluted 1:50, overnight at 4 °C. The day after, the sections were rinsed twice for 10 minutes in PBS and incubated with the second primary antibody (Glucagon K9bB10, code sc-57171, Santa Cruz Biotechnology, Europe) diluted 1:50, overnight at 4°C. After washing twice in PBS all sections were incubated with fluorescent secondary antibodies such us rabbit-IgG conjugated with fluorescein isothiocyanate (FITC; diluted 1:100; Sigma-Aldrich, Milan, Italy) and mouse IgG antibody conjugated with Texas Red (TRITC; diluted 1:100, Sigma-Aldrich, Milan, Italy) respectively. The nuclei were counterstained with Hoechst (Sigma-Aldrich, Inc, Milan, Italy) for 15 min at 23 °C. Finally, all slides were mounted with cover slips using a drop of PBS, and readings and imaging were immediately performed with a Leica DM5000 upright fluorescence microscope (Leica Microsystems, Heidelberg, Germany) connected to a high-resolution camera, Leica DC300 F (Leica). The cell positivity of both markers (“Merge”) was assessed through the program ImageJ software (ImageJ 1.43u, NIMH, Bethesda, Maryland, USA). The examination and the computer analysis of the histological sections were performed without knowledge of the origin of the tissue samples. For double immunofluorescence to detect macroH2A1.1/macroH2A1.2 in hearts and livers of C57BL/6 WT mice, tissues were cryo-preserved in OCT embedding matrix (Cellpath), snap frozen in liquid nitrogen (N2), and stored at -80˚C. Thin sections (8μm) were cut at -20ÚC with the help of a cryostat (Leica) and fixed with 4% Paraformaldehyde (PFA) (w/v) in PBS for 10 min. at RT, and then processed for blocking. Fat tissue was instead fixed with 4% Paraformaldehyde (PFA) (w/v) in PBS for 48h at 4C, and processed for paraffin embedding. Thin sections (8μm) where cut with the help of a microtome (Leica) and dried at 42ÚC for 24h. Upon sections deparaffinization and rehydratation, antigens were unmasked in antigen unmasking solution based on citrate buffer (Vector laboratories) and cooled in cold PBS 1X. For immunolabelling, sections of all tissues (liver, heart and adipose) were quenched for 10 min with 100mm NH_4_Cl, permeabilized for 15 min. with 0.5% (v/v) Triton X100 and blocked for 1h with (10% (w/v) BSA, 0.1% fish gelatine (Sigma) in PBS. Subsequently sections were incubated with primary antibodies [MacroH2A1.1, MacroH2A1.2 rabbit immunopurified sera [5]; anti-perilipin A/C (goat antibody, ABR Affinity BioReagents)] diluted 1/50 in 0.5% BSA, 0.1% gelatine, PBS (PBG) overnight at 4˚C and with secondary antibodies (Goat anti Rabbit-Alexa488 coniugated, Life Technologies; anti-goat IgG–FITC antibody produced in donkey, CF 488A, Sigma-Aldrich; anti-rabbit IgG–TRITC antibody produced in goat, T5268, Sigma-Aldrich) diluted 1:1000 in PBG for 2h at RT. Nuclei were visualised with DAPI or Hoechst coloration, and sections were mounted with Prolong medium (Life Technologies). Immunolabelled sections were observed with a laser scanning confocal microscope, TCS SP5 (Leica Microsystems, Mannheim). An Argon laser operating at 488 nm wavelength, and a UV 405 nm were used as excitation sources. Optical sections of 0.3 μm were acquired.

**Supplemental Table I.** Patient data – adipose tissue biopsies.

| **Patient ID** | **BMI** | **Age** | **Gender** | **Surgical Procedure** |  | **Pathology** |  |  |
| --- | --- | --- | --- | --- | --- | --- | --- | --- |
| **Patient 1** | 40 | 72 | F | Whipple’s procedure |  | Pancreatic adenocarcinoma |  |  |
| **Patient 2** | 28 | 73 | M | Right Hepatectomy |  | Colorectal liver metastases |  |  |
| **Patient 3** | 27 | 54 | F | Whipple’s procedure |  | Pancreatic adenocarcinoma |  |  |
| **Patient 4** | 26 | 67 | M | Distal Pancreatectomy, splenectomy and total gastrectomy |  | Neuroendocrine tumour of the pancreas |  |  |
| **Patient 5** | 30 | 66 | F | Wedge resection seg 6,7,8 of the liver |  | Colorectal liver metastases |  |  |
| **Patient 6** | 26 | 73 | M | Seg 3 liver resection |  | Colorectal liver metastases |  |  |
| **Patient 7** | 25 | 42 | M | Wedge resections Seg 8, 4b, 2, 4b/5 of the liver |  | Colorectal liver metastases |  |  |

**Supplemental Table II.** Sequences of primers amplifying promoter regions

**Gene Primers Region amplified (from TSS)**

**FASN**  1. Fw: CACGATGACCGGTAGTAACCC from -627 to -501

1. Rv: GGGCGTTGCTAGGCAATAGG

2. Fw: CCTATTGCCTAGCAACGCCC from -551 to -469

2. Rv: GGCCTGCTCCACATCGAAA

3. Fw: TGGAATCGCAGCGACACG from -455 to -385

3. Rv: ACTGGAAACTCTGCACTGAGG

4. Fw: GCGCACAGTGCACACCT from -355 to -109

4. Rv: CAATGAGCGTCGGGGC

5. Fw: AGCCCCGACGCTCATTG from -120 to -6

5. Rv: GCTATTTAAACCGCGGCCATC

6. Fw: GGGATGGCCGCGGTTTAAATA from -50 to +64

6. Rv: CTCTGGAGGCAGACGACAA

**SIRT1**  1. Fw: TCCTGGCCCTGTCATTTTAG from -816 to -623

1. Rv: GAAAGATCTGGCGATTGAGG

2. Fw: CTCAATCGCCAGATCTTTCC from -631 to -510

2. Rv: GCCTCAAGTCGGCTTTTACC

3. Fw: GAAGAGGCGCAGGCAGG from -490 to -456

3. Rv: CGGTGGTTCAAGTTTGCGAT

4. Fw: AGGCCCAGGTGGGAAGTG from -313 to -168

4. Rv: CACAACACGCCGGGTCAC

5. Fw: TTAAATCTCCCGCAGCCGAG from -111 to -85

5. Rv: TGCCTCTCTGGCCCTCC

**RETN**  1. Fw: CCTCCGTATGCACATCACAC from -886 to -628

1. Rv: TTCTGGTGCAAGGAGCAAG

2. Fw: TTGCACCAGAAAGCAGAAAG from -650 to -453

2. Rv: CTAGAGACCCAGCCCCTCTC

3. Fw: ACCACTGAGAGGCTGGAGAC from -424 to -212

3. Rv: GTAGGGCTGGAGATGCAAAC

4. Fw: CCAGCCCTACAGGTGAAGTC from -415 to -178

4. Rv: TTGTTGACTTCTGGCCCATC

5. Fw: TTATTATCCAAGGCAGGATGG from -132 to -20

5. Rv: ATCACTTACCGTGGCAGAGG

**E2F1**  1. Fw: TTGCAAACATCTGCTGCTTC from -954 to -756

1. Rv: GCCTTTAATCCCAGCACTTG

2. Fw: CTAACTGGGCTCTGGCTACG from --718 to -513

2. Rv: CTTCAGGCTCACCTCCAAAG

3. Fw: ACGTGCAGAACCGAGTACG from -432 to -306

3. Rv: CTTCTCCCCTCTGCCTCTC

4. Fw: AGAGGCAGAGGGGAGAAGG from -326 to -87

4. Rv: AAAGTCCGGGCCACTTTTAC

Supplemental Table III

| |  | **Up-Down Regulation (comparing to control group - WT mice)** |  | | |  | | --- | --- | --- | --- | --- | --- | |  |  | **WT+HF** | **TG** | **TG+HF** |  | | **Refseq** | **Symbol** | **Fold Regulation** | **Fold Regulation** | **Fold Regulation** |  | | NM_133904 | Acacb | -2.152 | 2.043 | 4.166 |  | | NM_007428 | Agt | 3.625 | 1.675 | -7.189 |  | | NM_007426 | Angpt2 | 19.366 | 2.223 | -12.267 |  | | NM_009733 | Axin1 | -10.932 | 1.056 | 2.339 |  | | NM_007557 | Bmp7 | 2.009 | 1.564 | 4.168 |  | | NM_007631 | Ccnd1 | -4.685 | 1.678 | 9.016 |  | | NM_009870 | Cdk4 | -3.609 | 1.503 | 5.552 |  | | NM_007669 | Cdkn1a | -2.792 | 1.676 | 6.039 |  | | NM_009875 | Cdkn1b | -2.537 | 1.354 | 3.576 |  | | NM_007891 | E2f1 | -5.803 | 1.024 | 9.323 |  | | NM_010118 | Egr2 | -13.562 | 1.905 | 51.635 |  | | NM_007988 | Fasn | 10.948 | 1.678 | -12.389 |  | | NM_010591 | Jun | -3.086 | 1.204 | 10.184 |  | | NM_019390 | Lmna | -3.561 | 0.976 | 6.853 |  | | NM_022984 | Retn | 6.075 | 1.054 | -2.182 |  | | NM_019812 | Sirt1 | -2.116 | 1.114 | 2.630 |  | | NM_022432 | Sirt2 | -2.101 | 1.265 | 3.814 |  | | NM_009204 | Slc2a4 | 6.788 | 1.545 | -4.585 |  | | NM_009463 | Ucp1 | -5.107 | 1.354 | 4.051 |  | | NM_009525 | Wnt5b | 2.800 | 1.205 | 2.618 |  | |
| --- | --- | --- | --- | --- | --- | --- | --- | --- | --- | --- | --- | --- | --- | --- | --- | --- | --- | --- | --- | --- | --- | --- | --- | --- | --- | --- | --- | --- | --- | --- | --- | --- | --- | --- | --- | --- | --- | --- | --- | --- | --- | --- | --- | --- | --- | --- | --- | --- | --- | --- | --- | --- | --- | --- | --- | --- | --- | --- | --- | --- | --- | --- | --- | --- | --- | --- | --- | --- | --- | --- | --- | --- | --- | --- | --- | --- | --- | --- | --- | --- | --- | --- | --- | --- | --- | --- | --- | --- | --- | --- | --- | --- | --- | --- | --- | --- | --- | --- | --- | --- | --- | --- | --- | --- | --- | --- | --- | --- | --- | --- | --- | --- | --- | --- | --- | --- | --- | --- | --- | --- | --- | --- | --- | --- | --- | --- | --- | --- | --- | --- | --- | --- | --- | --- | --- | --- | --- | --- |
|  |
|  |
|  |
| Values represent means of fold regulation of 3 mice per condition (WT + HF diet, TG, TG + HF diet) compared to control (WT animals).  In red = genes upregulated > 2 fold compared to wild type mice  In blue = genes downregulated > 2 fold compared to wild type mice |
|  |
|  |
|  |
|  |
|  |
|  |
|  |
|  |
|  |
|  |
|  |
|  |
|  |
|  |
|  |
|  |
|  |

**Supplemental References**

1. Vinciguerra M, Veyrat-Durebex C, Moukil MA, Rubbia-Brandt L, Rohner-Jeanrenaud F, Foti M: **PTEN down-regulation by unsaturated fatty acids triggers hepatic steatosis via an NF-kappaBp65/mTOR-dependent mechanism**. *Gastroenterology* 2008, **134**(1):268-280.

2. Benegiamo G, Mazzoccoli G, Cappello F, Rappa F, Scibetta N, Oben J, Greco A, Williams R, Andriulli A, Vinciguerra M *et al*: **Mutual antagonism between circadian protein period 2 and hepatitis C virus replication in hepatocytes**. *PLoS One* 2013, **8**(4):e60527.

3. Kleiner DE, Brunt EM, Van Natta M, Behling C, Contos MJ, Cummings OW, Ferrell LD, Liu YC, Torbenson MS, Unalp-Arida A *et al*: **Design and validation of a histological scoring system for nonalcoholic fatty liver disease**. *Hepatology* 2005, **41**(6):1313-1321.

4. Mathur A, Marine M, Lu D, Swartz-Basile DA, Saxena R, Zyromski NJ, Pitt HA: **Nonalcoholic fatty pancreas disease**. *HPB (Oxford)* 2007, **9**(4):312-318.

5. Rappa F, Greco A, Podrini C, Cappello F, Foti M, Bourgoin L, Peyrou M, Marino A, Scibetta N, Williams R *et al*: **Immunopositivity for histone macroH2A1 isoforms marks steatosis-associated hepatocellular carcinoma**. *PLoS One* 2013, **8**(1):e54458.
