# Supplementary figures and images for: Histone macroH2A1.2 promotes metabolic health and leanness by inhibiting adipogenesis
Source: Epigenetics Chromatin. 2016 Oct 25;9:45. doi: 10.1186/s13072-016-0098-9 (PMC5078890; doi:10.1186/s13072-016-0098-9)

## Slide 1
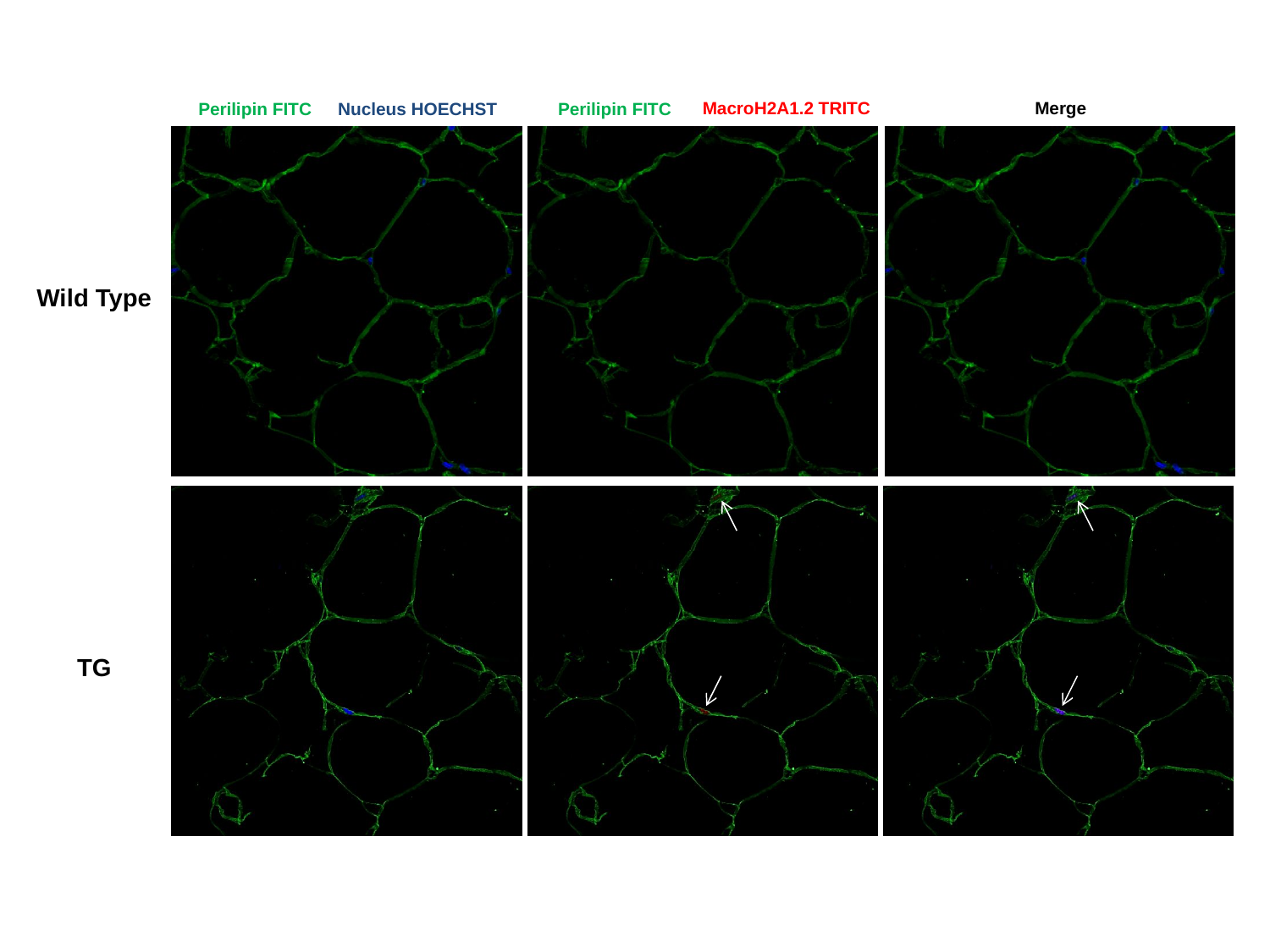

MacroH2A1.2 TRITC
Merge
Nucleus HOECHST
Perilipin FITC
Perilipin FITC
Wild Type
TG

Supplement: Supplementary file 2 — Additional file 2. Figure S1. Representative images of wild-type and macroH2A1.2 transgenic (Tg) mice adipose tissue (VAT) sections immunostained for macroH2A1.2 (red). Nuclei were counterstained with Hoechst (blue), while perilipin was immunostained to define adipose cell membranes (green). macroH2A1.2 expression was detected only in the VAT of Tg animals but not in wild-type animals (white arrows). [file 13072_2016_98_MOESM2_ESM.pptx]

## Slide 1
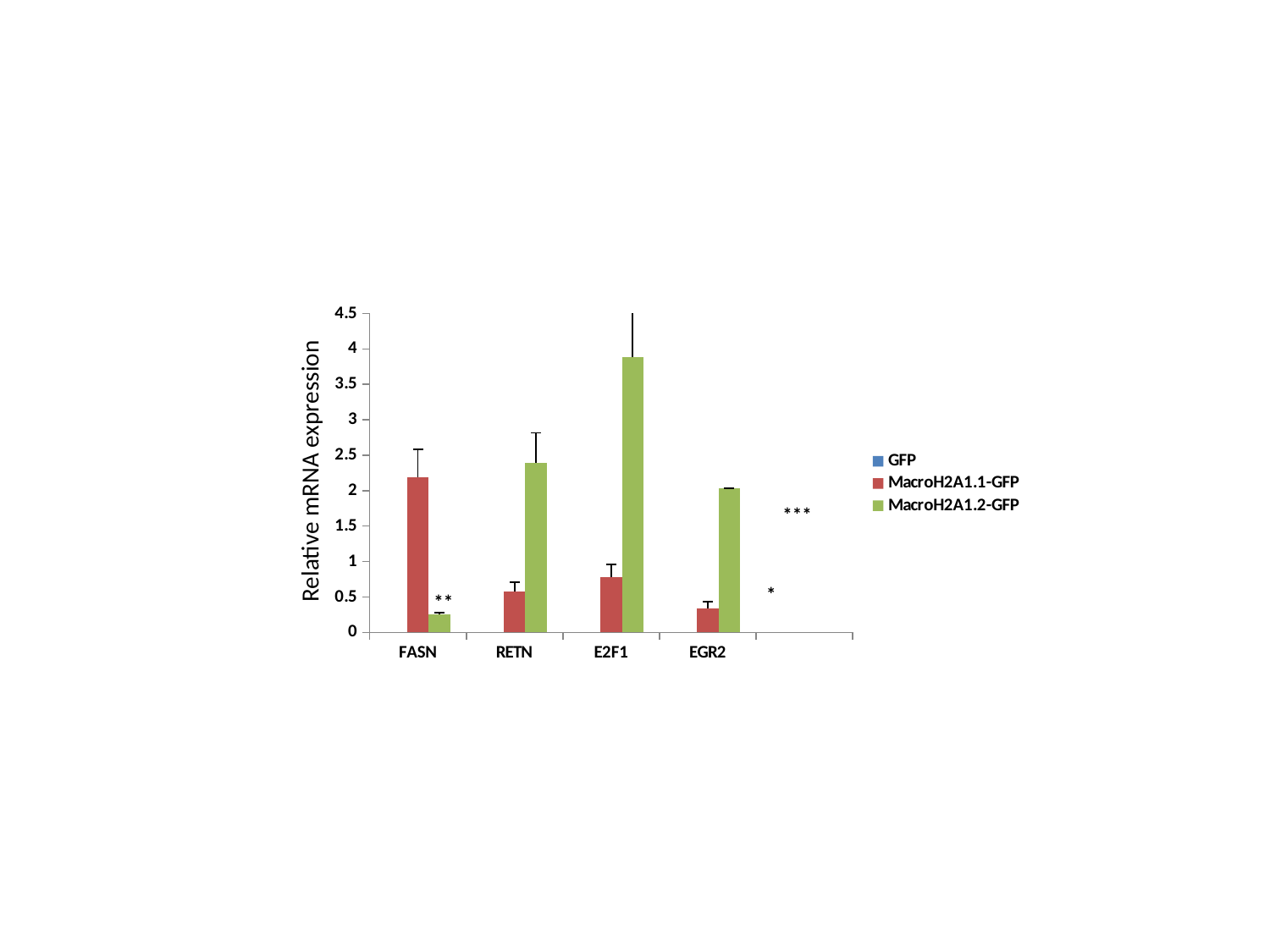

### Chart
| Category | GFP | MacroH2A1.1-GFP | MacroH2A1.2-GFP |
|---|---|---|---|
| FASN | 1.0 | 2.19 | 0.25 |
| RETN | 1.0 | 0.576666666666667 | 2.396666666666667 |
| E2F1 | 1.0 | 0.783333333333333 | 3.883333333333333 |
| EGR2 | 1.0 | 0.343333333333333 | 2.03 |Relative mRNA expression
***
*
**

Supplement: Supplementary file 5 — Additional file 5. Figure S4. Gene expression in 3T3-L1 adipocytes. 3T3-L1 pre-adipocytes with lentiviral-mediates stable expression of GFP, macroH2A1.1-GFP and macroH2A1.2-GFP were induced to differentiate into mature adipocytes as in Fig. 6. At the 15th day of differentiation, RNA was extracted and processed for qPCR analyses with specific primers. Results were normalized to pre-differentiation gene levels. Values are represented as means (N = 3) ± S.E.M. *P < 0.05; ***P < 0.0001 change vs GFP. [file 13072_2016_98_MOESM5_ESM.pptx]

## Slide 1
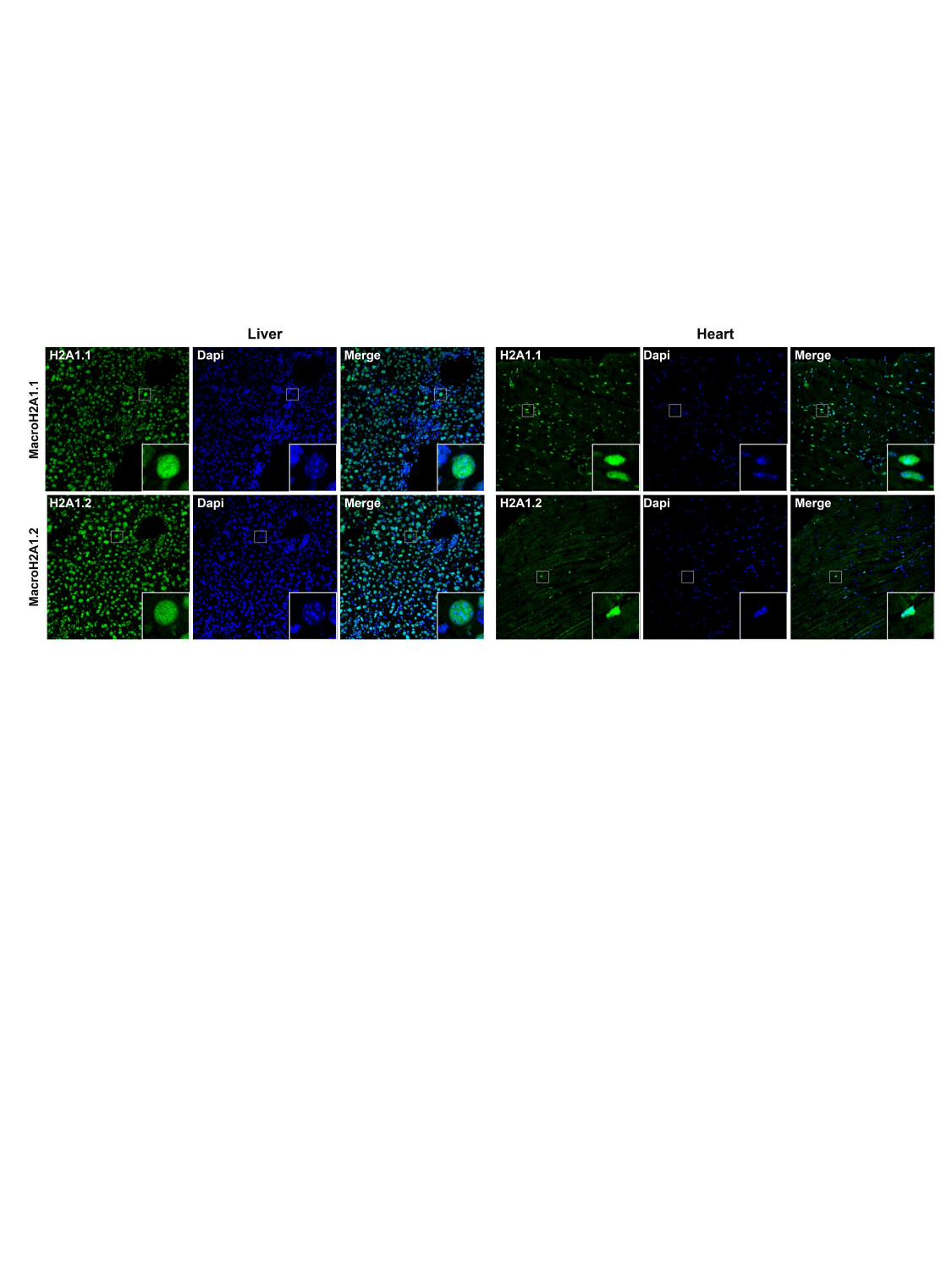

Supplement: Supplementary file 6 — Additional file 6. Figure S5. Representative images of liver (left panels) and heart (right panels) sections immunostained for macroH2A1.1 or for macroH2A1.2 (green). Both isoforms appear to be highly expressed in hepatocytes, whereas there a strong reduction in expression pattern of macroH2A1.2 is observed in mouse heart tissue. Nuclei were counterstained with DAPI. [file 13072_2016_98_MOESM6_ESM.pptx]

## Slide 1
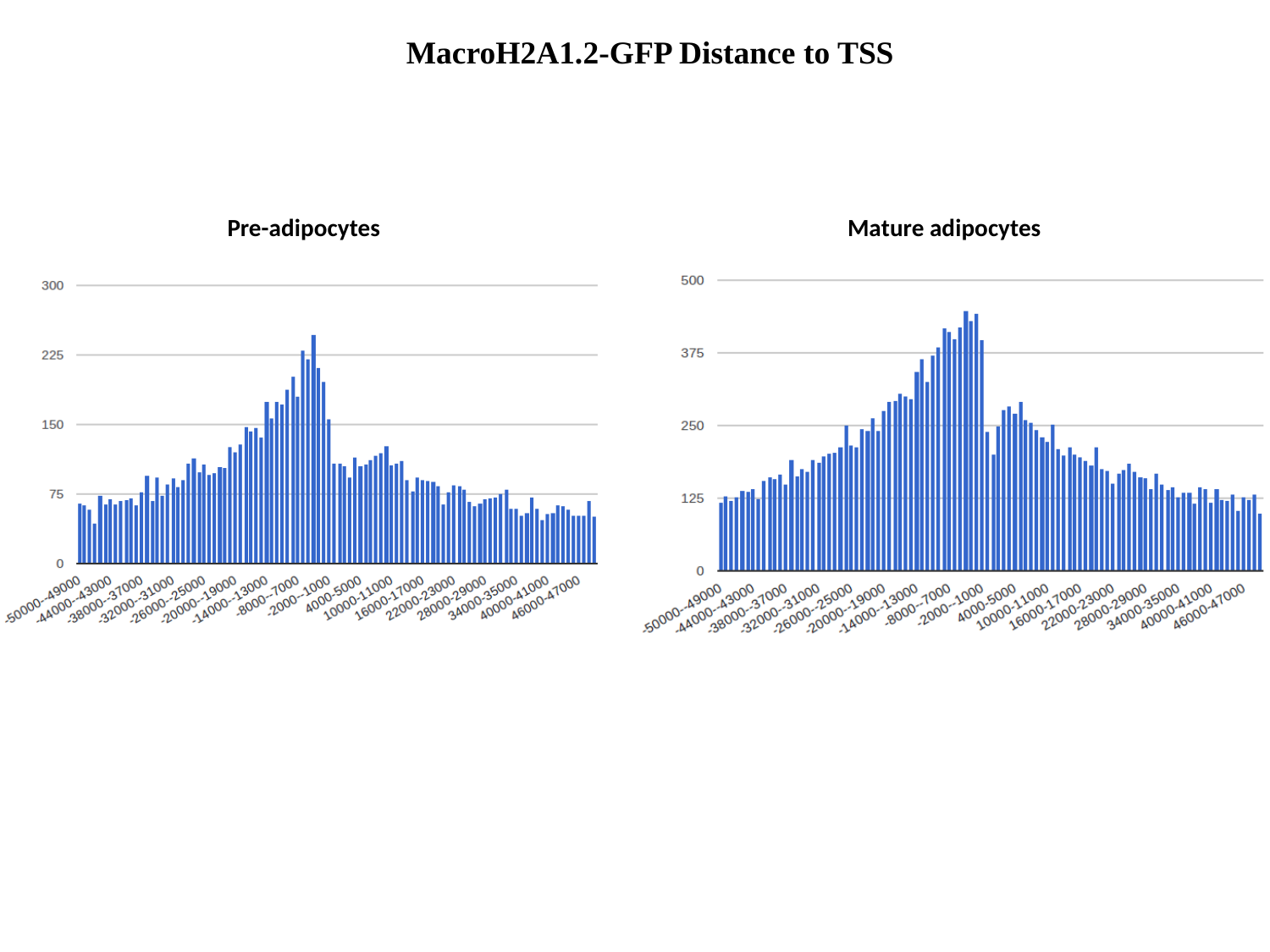

MacroH2A1.2-GFP Distance to TSS
Pre-adipocytes
Mature adipocytes

Supplement: Supplementary file 7 — Additional file 7. Figure S6. Histogram representing the distance and the frequency of macroH2A1.2-binding regions from transcriptional starting site (TSS), genome-wide. [file 13072_2016_98_MOESM7_ESM.pptx]
