# Supplementary material for: Histone macroH2A1.2 promotes metabolic health and leanness by inhibiting adipogenesis
Source: Epigenetics Chromatin. 2016 Oct 25;9:45. doi: 10.1186/s13072-016-0098-9 (PMC5078890; doi:10.1186/s13072-016-0098-9)
Supplement: Supplementary file 3 — Additional file 3. Figure S2. Increased glucose clearance because of enhanced insulin sensitivity in the muscle, liver and adipose tissue. Mice fed a chow diet were injected with insulin (INS, 0.75 U kg − 1) 15 min before being killed, after which phosphorylation status of AKT (Ser473) was determined by western blot. Representative immunoblots are shown in the skeletal muscle, liver and adipose tissue. Immunoblots were quantified by densitometry and normalized against total protein levels of AKT. *P < 0.05, ***P < 0.001 change WT + INS vs WT; ###P < 0.001 change Tg + INS vs Tg; $P < 0.05 Tg + INS vs WT + INS. [file 13072_2016_98_MOESM3_ESM.pptx]

## Slide 1
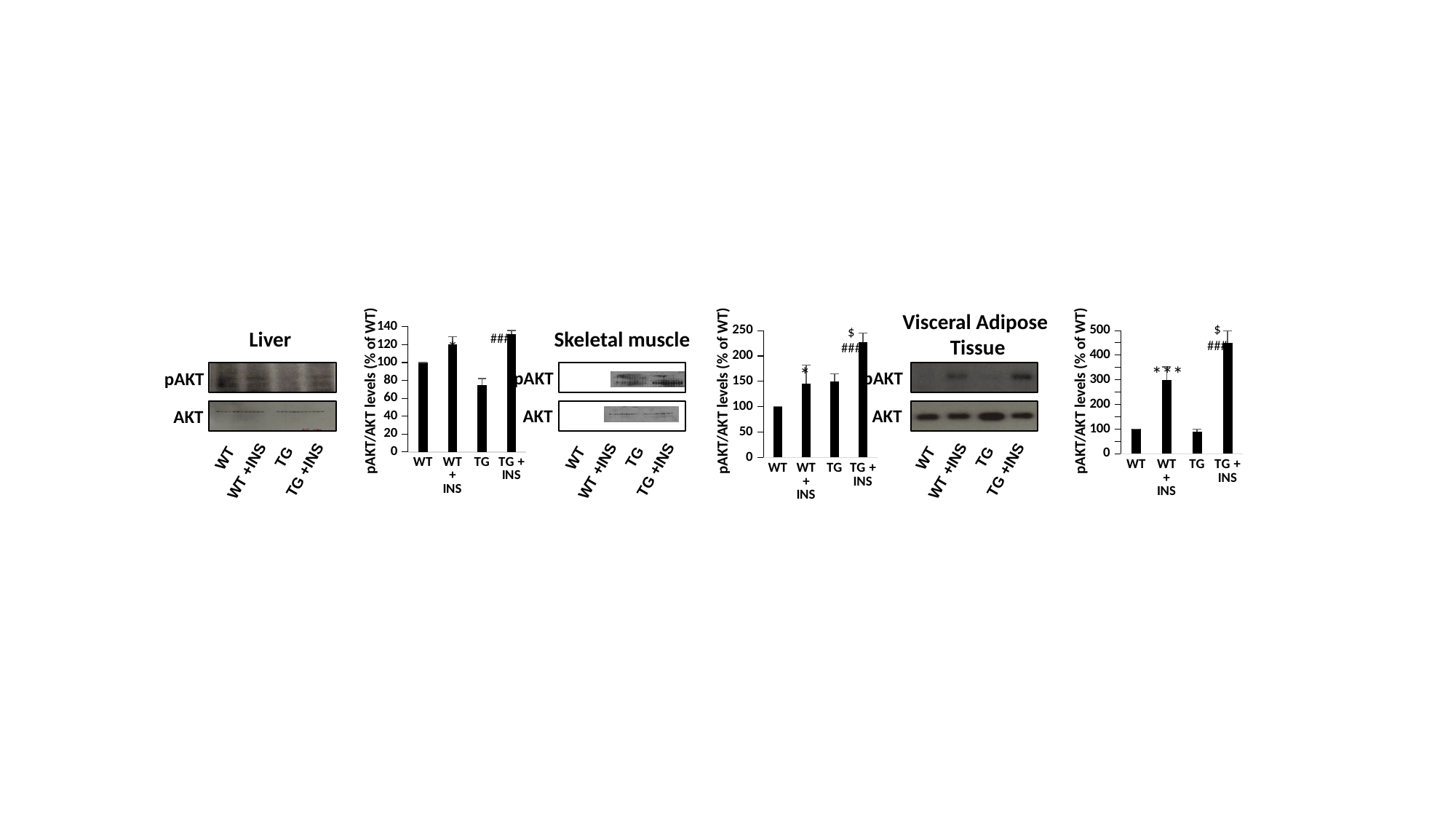

Visceral Adipose
Tissue
pAKT/AKT levels (% of WT)
pAKT/AKT levels (% of WT)
pAKT/AKT levels (% of WT)
$
###
$
###
Liver
### Chart
| Category | pAKT/AKT |
|---|---|
| WT | 100.0 |
| WT + INS | 120.0 |
| TG | 75.0 |
| TG + INS | 132.0 |Skeletal muscle
### Chart
| Category | pAKT/AKT |
|---|---|
| WT | 100.0 |
| WT + INS | 145.0 |
| TG | 150.0 |
| TG + INS | 227.0 |
### Chart
| Category | pAKT/AKT |
|---|---|
| WT | 100.0 |
| WT + INS | 300.0 |
| TG | 90.0 |
| TG + INS | 450.0 |###
*
***
*
pAKT
pAKT
pAKT
AKT
AKT
AKT
TG
TG
TG
WT
WT
WT
TG +INS
TG +INS
TG +INS
WT +INS
WT +INS
WT +INS
