# Supplementary material for: Histone macroH2A1.2 promotes metabolic health and leanness by inhibiting adipogenesis
Source: Epigenetics Chromatin. 2016 Oct 25;9:45. doi: 10.1186/s13072-016-0098-9 (PMC5078890; doi:10.1186/s13072-016-0098-9)
Supplement: Supplementary file 4 — Additional file 4. Figure S3. MacroH2A1.1 and macroH2A1.2 expression in 3T3-L1 pre-adipocytes and adipocytes. 3T3-L1 pre-adipocytes with lentiviral-mediates stable expression of GFP, macroH2A1.1-GFP and macroH2A1.2-GFP were induced to differentiate into mature adipocytes as in Fig. 6. At the 1st, 5th and 15th day of differentiation, histones were extracted and processed for immunoblotting with anti-macroH2A1.1, macroH2A1.2 and anti-H3-specific antibodies. Representative blots are shown, together with MW ladder. [file 13072_2016_98_MOESM4_ESM.pptx]

## Slide 1
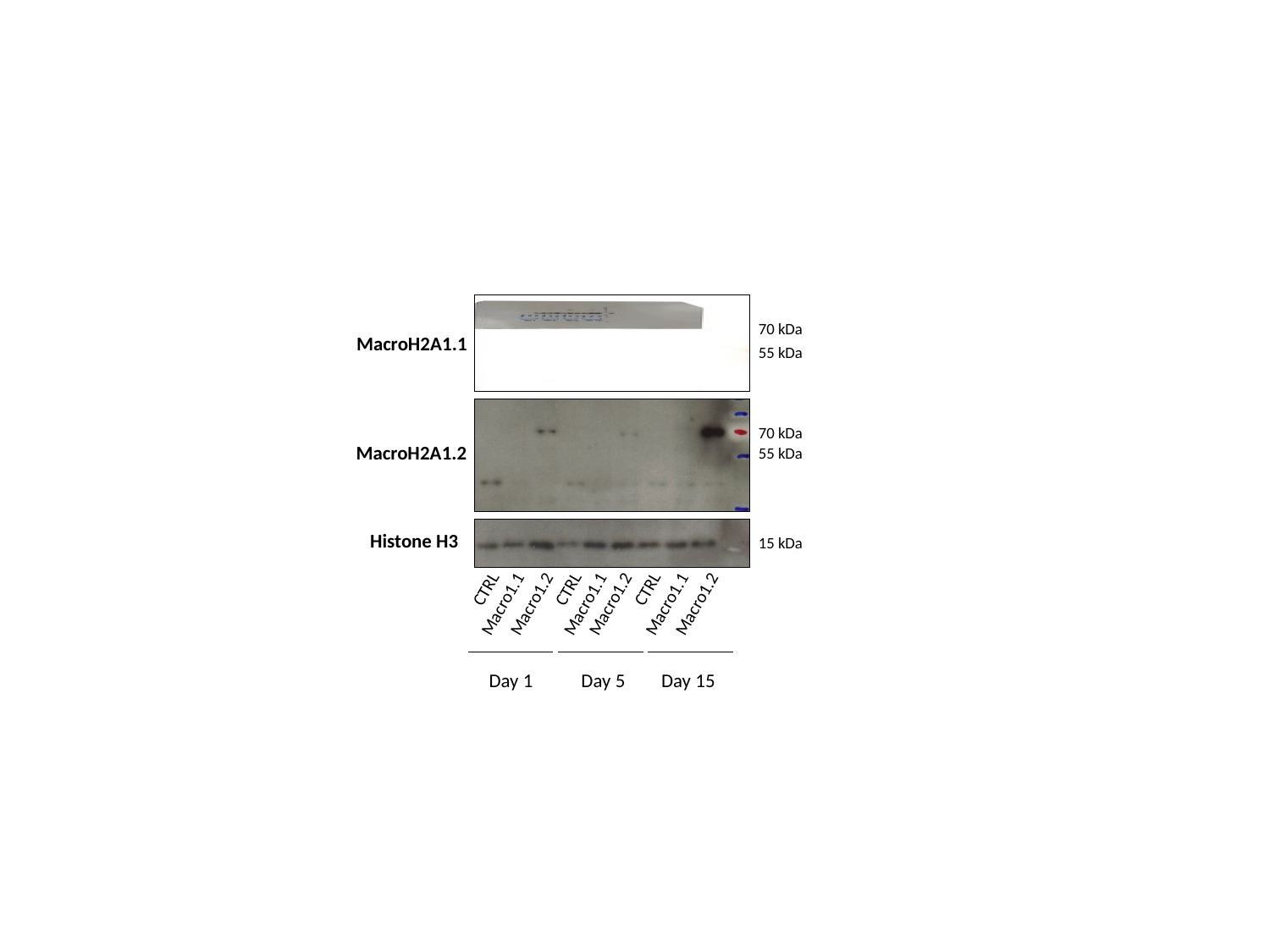

70 kDa
MacroH2A1.1
55 kDa
70 kDa
MacroH2A1.2
55 kDa
Histone H3
15 kDa
CTRL
CTRL
CTRL
Macro1.1
Macro1.2
Macro1.1
Macro1.2
Macro1.1
Macro1.2
Day 5
Day 1
Day 15
